# Supervised versus unsupervised primaquine radical cure for the treatment of falciparum and vivax malaria in Papua, Indonesia: a cluster-randomised, controlled, open-label superiority trial

**DOI:** 10.1016/S1473-3099(21)00358-3

**Published:** 2022-03

**Authors:** Jeanne Rini Poespoprodjo, Faustina Helena Burdam, Freis Candrawati, Benedikt Ley, Niamh Meagher, Enny Kenangalem, Ratni Indrawanti, Leily Trianty, Kamala Thriemer, David J Price, Julie A Simpson, Ric N Price

**Affiliations:** a Centre for Child Health and Department of Child Health, Faculty of Medicine, Public Health and Nursing, Universitas Gadjah Mada, Yogyakarta, Indonesia; b Timika Malaria Research Facility, Papuan Health and Community Development Foundation, Timika, Papua, Indonesia; c Mimika District Hospital and District Health Authority, Timika, Papua, Indonesia; d Global Health Division, Menzies School of Health Research and Charles Darwin University, Darwin, NT, Australia; e Centre for Epidemiology and Biostatistics, Melbourne School of Population and Global Health, University of Melbourne, VIC, Australia; f Victorian Infectious Diseases Reference Laboratory Epidemiology Unit at the Peter Doherty Institute for Infection and Immunity, University of Melbourne and Royal Melbourne Hospital, VIC, Australia; g Eijkman Institute for Molecular Biology, Jakarta, Indonesia; h Centre for Tropical Medicine, Nuffield Department of Clinical Medicine, University of Oxford, Oxford, UK; i Mahidol-Oxford Tropical Medicine Research Unit (MORU), Faculty of Tropical Medicine, Mahidol University, Bangkok, Thailand

## Abstract

**Background:**

There is a high risk of *Plasmodium vivax* recurrence in patients treated for *Plasmodium falciparum* malaria in co-endemic areas. Primaquine radical cure has the potential to reduce *P vivax* recurrences in patients presenting with *P falciparum* as well as *P vivax* malaria but is undermined by poor adherence to the currently recommended 14-day regimen. We aimed to assess the efficacy and safety of supervised versus unsupervised primaquine radical cure in patients presenting with uncomplicated malaria.

**Methods:**

We did a cluster-randomised, controlled, open-label superiority trial in Papua, Indonesia. 21 clusters of village health posts, matched by annual parasite index, were randomly assigned (1:1) to treat patients (age >12 months and body weight >5 kg) presenting with confirmed uncomplicated *P falciparum* or *P vivax* malaria with oral dihydroartemisinin–piperaquine plus either a supervised or unsupervised 14-day course of oral primaquine (0·5 mg/kg per day). Patients in the supervised group were supervised taking their primaquine dose on alternate days. Patients were followed-up for 6 months and those who presented again with malaria were retreated with the same drug regimen. Masking was not possible due to the nature of the study. The primary outcome was the incidence risk of *P vivax* malaria over 6 months, assessed in the modified intention-to-treat population (all patients who were assigned to a treatment group, excluding patients who were lost to follow-up after their first visit). This trial is now complete, and is registered with ClinicalTrials.gov, NCT02787070.

**Findings:**

Between Sept 14, 2016, and July 31, 2018, 436 patients were screened for eligibility and 419 were enrolled; 223 (53%) patients in 11 clusters were assigned to supervised primaquine treatment and 196 (47%) in ten clusters to unsupervised primaquine treatment. 161 (72%) of 223 patients in the supervised group and 151 (77%) of 196 in the unsupervised group completed 6 months of follow-up. At 6 months, the incidence risk of *P vivax* recurrence in the supervised group was 29·7% (95% CI 16·4–49·9) versus 55·8% (32·3–81·8) in the unsupervised group (hazard ratio 0·23 [95% CI 0·07–0·76]; p=0·016). The incidence rate for *P vivax* recurrence was 539 (95% CI 390–747) infections per 1000 person-years in the supervised group versus 859 (673–1096) in the unsupervised group (incidence rate ratio 0·63 [95% CI 0·42–0·94]; p=0·025). The corresponding rates in the 224 patients who presented with *P falciparum* malaria were 346 (95% CI 213–563) and 660 (446–977; incidence rate ratio 0·52 [95% CI 0·28–0·98]; p=0·043). Seven serious adverse events were reported (three in the supervised group, four in the unsupervised group), none of which were deemed treatment-related, and there were no deaths.

**Interpretation:**

In this area of moderate malaria transmission, supervision of primaquine radical cure treatment reduced the risk of *P vivax* recurrence. This finding was apparent for patients presenting with either *P falciparum* or *P vivax* malaria. Further studies are warranted to investigate the safety and efficacy of radical cure for patients presenting with uncomplicated falciparum malaria in other co-endemic areas.

**Funding:**

The Bill & Melinda Gates Foundation, Wellcome Trust, and Department of Foreign Affairs and Trade of the Australian Government.

**Translation:**

For the Indonesian translation of the abstract see Supplementary Materials section.

## Introduction

There are between 7·5 million and 14·5 million cases of *Plasmodium vivax* malaria reported worldwide each year from 49 endemic countries.^1^ Outside of sub-Saharan Africa, *P vivax* is the predominant cause of malaria.^2^ *P vivax* is more difficult to eliminate than *Plasmodium falciparum*, because it forms dormant liver stages (hypnozoites) that can reactivate weeks to months after an initial infection, causing recurrent symptomatic illness (relapses). The risk and frequency of relapse varies considerably with geographical location.^3^ Frequent recurrent *P vivax* parasitaemia results in a cumulative risk of anaemia, and both direct and indirect morbidity and mortality, particularly in young children and pregnant women.^4^, ^5^, ^6^ Relapsing infections also sustain ongoing transmission of the parasite.^7^

**Research in context**

**Evidence before this study**

We searched PubMed, MEDLINE, Web of Science, Embase, and the Cochrane Database of Systematic Reviews from Jan 1, 1960, to Feb 28, 2021, for relevant clinical trials and systematic reviews, published in English, on the risk of *Plasmodium vivax* recurrence following *Plasmodium falciparum* infections, using the terms “vivax”, “falciparum”, and “recurrence”. The risk of *P vivax* recurrence within 63 days of treatment for *P falciparum* malaria exceeded 15% across a range of co-endemic areas, highlighting a potential benefit of primaquine radical cure for *P falciparum* malaria. The risk of *P vivax* recurrence within 12 months was less than 10% following supervised high-dose primaquine (total dose 7 mg/kg) treatment plus dihydroartemisinin–piperaquine, but increased to more than 88% when primaquine treatment was unsupervised. As of Feb 28, 2021, no trials were identified that quantified the efficacy or effectiveness of supervised 14-day primaquine or single-dose tafenoquine for the treatment of both vivax and falciparum malaria.

**Added value of this study**

This cluster-randomised, controlled, open-label, superiority trial showed that supervision of primaquine radical cure treatment on alternate days reduced the risk of *P vivax* recurrence in patients presenting with either *P vivax* or *P falciparum* malaria.

**Implications of all the available evidence**

In areas of moderate malaria transmission, active measures to ensure patient adherence to a complete course of primaquine radical cure treatment, and extending its use to all patients presenting with uncomplicated malaria due to either *P vivax* or *P falciparum*, has potential to reduce the risk of recurrent *P vivax* parasitaemia, which could reduce ongoing transmission and facilitate malaria elimination.

The radical cure of malaria refers to a combination of drugs to kill both the blood and liver stages of the parasite. Primaquine, an 8-aminoquinoline, is currently the only widely available drug that can kill *P vivax* liver stages and prevent relapse. Supervised administration of artemisinin-based combination therapy and a high dose of primaquine reduces the risk of recurrence, even in areas with high relapse periodicity,^8^ but its use is limited by the risk of severe haemolysis in patients with glucose-6-phosphate dehydrogenase (G6PD) deficiency. To improve its tolerability, the total dose of primaquine is usually spread over 14 days; however, unsupervised adherence to such a prolonged regimen is poor.^9^

Radical cure of malaria is currently restricted to patients presenting with *P vivax* or *Plasmodium ovale*, the only species causing human malaria that form hypnozoites.^10^ However, systematic reviews and meta-analyses have highlighted a high risk of *P vivax* parasitaemia in patients following treatment for *P falciparum* malaria.^11^, ^12^ We hypothesised that recurrent parasitaemia with *P vivax* originated from reactivation of occult hypnozoites in patients exposed to both species, potentially triggered by the acute febrile illness of *P falciparum* malaria.^12^ In view of this hypothesis, extending the indication for primaquine radical cure to patients presenting with all species of malaria has potential to reduce recurrent malaria and accelerate malaria elimination in co-endemic regions.^11^, ^12^, ^13^ We aimed to assess the efficacy and safety of high-dose primaquine administered over 14 days with supervision on alternate days versus the current practice of unsupervised treatment, in patients presenting with uncomplicated malaria due to either *P vivax* or *P falciparum* monoinfection or a mixed infection with both species.

## Methods

### Study design

We did a cluster-randomised, controlled, open-label superiority trial in Papua, Indonesia. The climate, geography, malaria endemicity, and demographics of the study site have been described previously.^14^ In brief, the study area lies in the lowland area of south-central Papua, and has perennial malaria transmission with 51% of malaria attributable to *P falciparum* infection, 45% to *P vivax*, and the remainder due to *Plasmodium malariae* or mixed species infections.^14^, ^15^

The unit of randomisation was the village health post. 21 village health posts (clusters) were included in the study and randomly assigned to one of the study treatment groups. Each health post was associated with one of eight public clinics where initial recruitment and treatment occurred. Clinics were selected to be located within 1·5 h drive from the research office and from those that treated more than 200 cases of malaria per year (appendix 2 p 2).

Written informed consent, or consent from the legal guardian, was required for participation. Ethical approval was obtained from the Human Research Ethics Committee of the Northern Territory Department of Health, Australia (HREC 15.2517) and the Health Research Ethics Committees of the University of Gadjah Mada, Indonesia (KE/FK/522/EC/2016).

### Participants

Febrile patients living in one of the study cluster villages and attending one of the eight public clinics with suspected malaria were screened for malaria by microscopic examination of Giemsa-stained peripheral blood film by a laboratory microscopist at the clinics. All slides were re-read the same day by an expert trial microscopist. Patients with confirmed uncomplicated *P falciparum* or *P vivax* malaria (including either monoinfection or mixed infections), who were older than 12 months and with body weight greater than 5 kg, were eligible for enrolment. Patients were excluded if they were pregnant (determined by history and urinary β-human chorionic gonadotropin test), lactating, G6PD deficient (determined by fluorescent blood spot test; Trinity Biotech, Bray, Ireland), anaemic (haemoglobin <9 g/dL), or had signs or symptoms of severe malaria using the criteria defined by WHO.^16^ Other exclusion criteria were hypersensitivity to any of the study drugs, or concomitant medication with potential to cause haemolysis or interfere with the pharmacokinetics of the study drugs. Patients who were diagnosed with G6PD deficiency were given a medical card and referred to their family clinician for management according to local guidelines.

### Randomisation and masking

The 21 clusters were selected according to location, size, and malaria transmission. Individual clusters with similar annual parasite index were then randomly assigned (1:1) to either the supervised or unsupervised primaquine treatment groups using Stata version 15.1. The independent statistician who generated the randomisation list and allocated clusters to treatment groups was not otherwise involved in the conduct of the trial. Masking was not possible due to the nature of the study.

### Procedures

After obtaining informed consent, eligible patients were enrolled and a baseline clinical questionnaire and examination were completed at the public clinic. All participants were immediately commenced on a 3-day regimen of oral dihydroartemisinin–piperaquine provided by the national malaria control programme according to national guidelines (appendix 2 p 3). All participants were reviewed on days 1 and 2 at the village health posts or at home, to ensure supervised schizontocidal treatment and symptom recovery. On day 2, participants had a repeat fingerprick test for haemoglobin concentration; if this was 9 g/dL or higher they were prescribed primaquine according to the study protocol.

Patients residing in the clusters assigned to unsupervised treatment were prescribed a course of oral primaquine according to local treatment guidelines, at a daily dose of 0·5 mg/kg per day, and were instructed to take the prescribed tablets once daily for 14 days (appendix 2 p 3). In the clusters assigned to supervised treatment, patients were prescribed the same primaquine regimen, but were visited on alternate days by a home visitor who provided them with primaquine tablets for that day and the following day. On day 16, all patients were instructed to either return to the village health post or were visited at home for clinical review. Patients in the unsupervised group were asked to return any remaining primaquine tablets for a pill count. In the supervised group, primaquine adherence was assessed by direct observation on the days of supervised administration and self-reported adherence for the previous unsupervised day. In the unsupervised group, adherence to primaquine was estimated from the pill count on day 16. Thereafter, all participants were followed-up on day 28 and then monthly for 6 months.

At each study visit, a medical history and symptom questionnaire were completed and any adverse events or serious adverse events were recorded by a research nurse, and referred to the study clinician as necessary. Patients were encouraged to present to the study centre if they became unwell at any time during the study. At each routine review or upon presentation with symptoms compatible with malaria, a capillary blood sample was taken for peripheral blood film examination and measurement of haemoglobin concentration (HemoCue Hb 201^+^; HemoCue, Ängelholm, Sweden). Blood film microscopy during follow-up was done by the study laboratory technicians, who were masked to treatment allocation.

Patients who presented with recurrent episodes of malaria were treated with the same treatment allocation as at enrolment and follow-up continued for a total of 6 months from the day of enrolment.

### Outcomes

The primary endpoint was the incidence risk of the first recurrent episode of *P vivax* parasitaemia over 6 months in patients with uncomplicated malaria due to either *P vivax* or *P falciparum* or both. Secondary endpoints were the incidence rate of all recurrent episodes of *P vivax* parasitaemia over 6 months in patients with uncomplicated malaria due to either *P vivax* or *P falciparum*, and the incidence risks and rates of *P vivax* malaria over 6 months in patients enrolled with *P vivax* or *P falciparum* seperately.

Safety endpoints were the proportion of patients vomiting within 1 h of administration of medication, vomiting of any primaquine dose in the supervised group during primaquine treatment, and adverse events or serious adverse events within 6 months in all patients. Haematological safety endpoints were the incidence risk of severe anaemia (haemoglobin <7 g/dL) or blood transfusion over 6 months, and an acute fall in haemoglobin greater than 5 g/dL or fractional fall of greater than 25% to a concentration of less than 7 g/dL within 14 days of starting primaquine treatment.

In a post-hoc analysis, the incidence risk and rate of *P falciparum* parasitaemia at 6 months was compared between supervised and unsupervised clusters.

### Statistical analysis

The required sample size was calculated to detect an absolute reduction of 20% in the incidence risk of *P vivax* recurrence over 6 months from 30% in the unsupervised group to 10% in the supervised group.^17^ Across the 21 clusters, a sample size of 420 participants (20 per cluster) provided 90% power to detect this difference with a two-sided significance level of 5%, assuming 15% loss to follow-up, and a conservative intracluster correlation coefficient of 0·05.^18^

The combined data from all clusters were analysed to provide a pragmatic comparison of the different treatments using a modified intention-to-treat strategy; analyses were done per the assigned treatment groups, regardless of whether participants were actually supervised or not, but excluding patients who were lost to follow-up after their first visit. Descriptive statistics of patient and disease characteristics at baseline were calculated by treatment group with frequency and percentage presented for categorical variables, and median with corresponding IQR for continuous variables. A clinically relevant decrease in haemoglobin was defined as an absolute decrease from baseline of more than 5 g/dL or a fractional fall of more than 25% to a concentration of less than 7 g/dL. Overall adherence to primaquine was evaluated by calculating the proportion of participants in each group who received a total dosage of at least 5 mg/kg of primaquine.

To maximise power, we did an unmatched analysis, as it was difficult to closely match all clusters on annual parasite index. Kaplan-Meier curves were produced to visualise the cumulative incidence risk of the first *P vivax* recurrence over 6 months in each treatment group. For the primary endpoint, hazard ratios (HRs) with 95% CIs were estimated using mixed-effects Cox proportional hazards regression with a time-varying coefficient for treatment effect (to account for non-proportional hazards) and a frailty term for clustering for time to first *P vivax* recurrence analyses. Incidence rate ratios (IRRs) were estimated using negative binomial regression with robust SE estimation (to account for clustering) for analysis of all *P vivax* recurrences. Details regarding censoring for the first *P vivax* recurrence, and safety analyses are provided in an a priori statistical plan (appendix 2 pp 4–7). All analyses were done using STATA version 15.1. This trial is registered with ClinicalTrials.gov, NCT02787070.

### Role of the funding source

The funders of the study had no role in study design, data collection, data analysis, data interpretation, or writing of the report.

## Results

**Figure 1:**
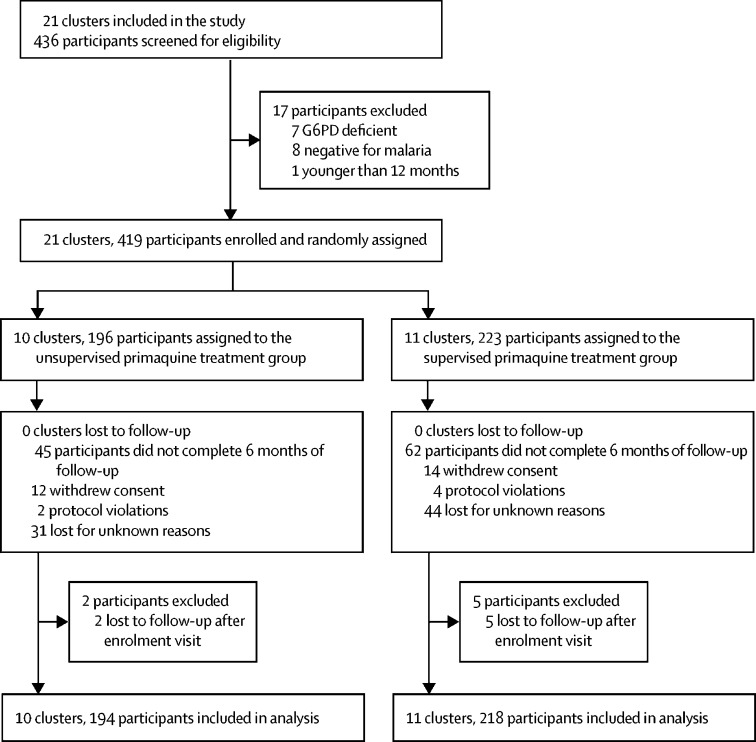
Trial profile G6PD=glucose-6-phosphate dehydrogenase.

Between Sept 14, 2016, and July 31, 2018, 436 patients were screened for eligibility and 419 were enrolled; 223 (53%) patients in 11 clusters were assigned to supervised primaquine treatment and 196 (47%) in ten clusters to unsupervised primaquine treatment (figure 1). At enrolment, 224 (53%) of 419 patients had *P falciparum* monoinfection, 183 (44%) had *P vivax* monoinfection, and 12 (3%) had a mixed species infection. Baseline characteristics were similar between the supervised and unsupervised treatment groups (table 1). Seven patients (five in the supervised group and two in the unsupervised group) were lost to follow-up immediately after their enrolment visit and were excluded from further analysis of recurrence outcomes.

**Table 1:** Baseline characteristics

		**Supervised group (n=223)**	**Unsupervised group (n=196)**	**Total (n=419)**
Sex*				
	Female	101 (45%)	94 (48%)	195 (47%)
	Male	122 (55%)	101 (52%)	223 (53%)
Age, years*		16·8 (7·8–32·3)	18·0 (7·4–35·5)	17·2 (7·4–33·1)
Age group, years				
	<5	39 (17%)	36 (18%)	75 (18%)
	5 to <15	59 (26%)	47 (24%)	106 (25%)
	≥15	120 (54%)	110 (56%)	230 (55%)
Weight, kg		46·9 (22·2–58·1)	48·3 (18·9–56·7)	47·8 (20·0–57·5)
Weight category, kg				
	<9	8 (4%)	6 (3%)	14 (3%)
	9 to <18	39 (17%)	37 (19%)	76 (18%)
	18 to <36	42 (19%)	32 (16%)	74 (18%)
	≥36	134 (60%)	121 (62%)	255 (61%)
Ethnicity*				
	Non-Papuan	128 (57%)	99 (51%)	227 (54%)
	Highland Papuan	56 (25%)	25 (13%)	81 (19%)
	Lowland Papuan	39 (17%)	71 (36%)	110 (26%)
History of malaria in the past 28 days				
	No	204 (91%)	184 (94%)	388 (93%)
	Yes	5 (2%)	1 (1%)	6 (1%)
	Unsure	14 (6%)	11 (6%)	25 (6%)
Species of infection				
	*Plasmodium falciparum*	120 (54%)	104 (53%)	224 (53%)
	*Plasmodium vivax*	97 (43%)	86 (44%)	183 (44%)
	Mixed infection	6 (3%)	6 (3%)	12 (3%)
Asexual *P falciparum* parasitaemia per μL blood*		4350 (750–12 900)	5738 (1275–11 738)	4931 (994–12 281)
Proportion with *P falciparum* parasitaemia		126 (57%)	110 (56%)	236 (56%)
Asexual *P vivax* parasitaemiaper μL blood		4538 (1838–10 425)	5063 (1125–11 888)	4688 (1313–11 063)
Proportion with *P vivax* parasitaemia		103 (46%)	92 (47%)	195 (47%)
Asexual *P falciparum* and *P vivax* parasitaemia per μL blood		9656 (2400–13 650)	14 156 (11 663–16 425)	12 544 (6750–15 656)
Gametocytaemia				
	Proportion with *P vivax* gametocytaemia	26 (27%)	31 (36%)	57 (31%)
	Proportion with *P falciparum* gametocytaemia	14 (12%)	11 (12%)	25 (11%)
	Proportion with *P vivax* and *P falciparum* gametocytaemia	2 (33%)	2 (33%)	4 (33%)
Temperature, °C*		36·6 (36·0–37·8)	36·8 (36·2–38·0)	36·7 (36·1–37·9)
Fever				
	<37·5°C	156 (70%)	125 (64%)	281 (67%)
	≥37·5°C	66 (30%)	71 (36%)	137 (33%)
Haemoglobin, g/dL		11·7 (10·2–13·7)	11·4 (10·3–12·9)	11·5 (10·3–13·3)

Data are n (%) or median (IQR).

* Missing data (for <2% observations)..

The initial response to schizontocidal treatment was rapid. Within 48 h, 399 (99%) of 405 patients had become afebrile and 375 (93%) had cleared their peripheral parasitaemia, with no significant difference in fever or parasite clearance between treatment groups. Clearance times could not be calculated in seven patients due to missed visit on day 2. On day 2 review, 213 (96%) of 223 patients in the supervised group and 191 (97%) of 196 in the unsupervised group with haemoglobin of at least 9 g/dL were prescribed primaquine (median total dose 7·4 mg/kg [range 3·5–15·4] and 7·4 mg/kg [4·3–14·2] respectively). During the period of primaquine treatment, 184 (83%) of 223 patients in the supervised group completed all required visits. 161 (72%) of 223 patients in the supervised group and 151 (77%) of 196 in the unsupervised group completed 6 months of follow-up.

**Figure 2:**
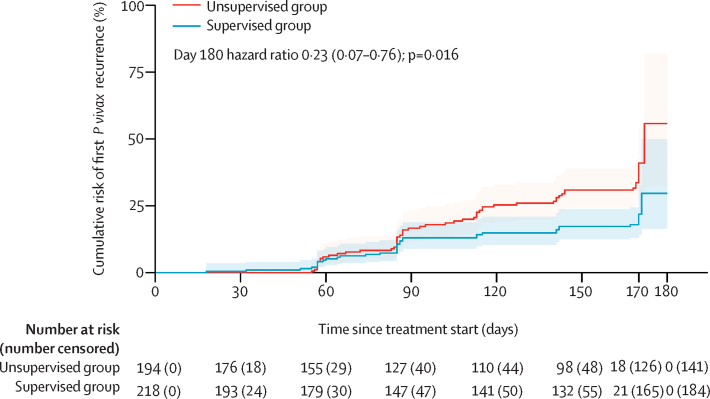
Cumulative incidence of the first recurrence of *Plasmodium vivax* parasitaemia The shaded areas represent 95% CIs.

At 6 months (day 180), the cumulative incidence risk of *P vivax* recurrence was 29·7% (95% CI 16·4–49·9) in the supervised group versus 55·8% (32·3–81·8) in the unsupervised group (figure 2, table 2). As the Kaplan-Meier survival curves crossed at approximately 60 days, the comparative risk of *P vivax* recurrence was estimated using a time-varying HR. At 6 months, the overall risk of *P vivax* recurrence was significantly lower in the supervised group than in the unsupervised group (HR 0·23 [95% CI 0·07–0·76]; p=0·016). The HR was 0·78 (95% CI 0·39–1·55; p=0·48) at day 60, and 0·43 (0·22–0·80; p=0·0084) at day 120.

**Table 2:** Cumulative incidence risk of first *Plasmodium vivax* recurrence and incidence rate of all *P vivax* recurrences during follow-up

	**Supervised group**	**Unsupervised group**	HR or IRR*	**p value**
**Cumulative incidence risk overall**				
Day 60	5·2% (2·8–9·5)	5·9% (3·2–10·7)	0·78 (0·39–1·55)	0·48
Day 120	14·9% (10·4–20·9)	25·3% (19·2–32·9)	0·43 (0·22–0·80)	0·0084
Day 180	29·7% (16·4–49·9)	55·8% (32·3–81·8)	0·23 (0·07–0·76)	0·016
**Incidence rate per 1000 person-years overall**				
Day 180	539 (390–747)	859 (673–1096)	0·63 (0·42–0·94)	0·025
**Cumulative incidence risk in patients who presented with *P vivax* (monoinfection or mixed infection)**				
Day 60	5·9% (2·5–13·5)	5·1% (2·0–13·1)	0·82 (0·33–2·08)	0·68
Day 120	19·6% (12·5–30·0)	37·1% (26·9–49·7)	0·35 (0·14–0·89)	0·027
Day 180	29·5% (16·9–48·2)	47·1% (33·9–62·5)	0·15 (0·02–0·95)	0·045
**Incidence rate per 1000 person-years in patients who presented with *P vivax* (monoinfection or mixed infection)**				
Day 180	778 (512–1182)	1095 (807–1485)	0·71 (0·42–1·20)	0·20
**Cumulative incidence risk in patients who presented with *Plasmodium falciparum* (monoinfection)**†				
Day 60	4·7% (2·0–11·0)	6·6% (3·0–14·1)	0·76 (0·31–1·89)	0·56
Day 120	11·0% (6·2–19·0)	16·0% (9·8–25·5)	0·50 (0·24–1·05)	0·068
Day 180	31·5% (11·0–70·8)	56·4% (26·1–89·7)	0·33 (0·08–1·44)	0·14
**Incidence rate (per 1000 person-years) in patients who presented with *P falciparum* (monoinfection)**†				
Day 180	346 (213–563)	660 (446–977)	0·52 (0·28–0·98)	0·043

Data are point estimates (95% CI) or p values. Number of recurrences and person-years observation for each cluster are presented in appendix 2 (p 9). HR=hazard ratio. IRR=incidence rate ratio.

* HRs are shown for cumulative incidence risks, IRRs are shown for incidence rates.

† Because of zero events in some clusters, it was not possible to include a shared frailty term for the Cox regression model in the *P falciparum* subgroup.

At day 28, four patients had recurrent parasitaemia (all in the supervised group), three of whom were infected with *P falciparum*, one with *P vivax*, and none with mixed infections. By 6 months, 95 patients had recurrence with *P falciparum*, 110 with *P vivax*, and six with mixed infections (appendix 2 p 8). The incidence rate for *P vivax* recurrence was 539 (95% CI 390–747) infections per 1000 person-years observed in the supervised group versus 859 (673–1096) in the unsupervised group (IRR 0·63 [95% CI 0·42–0·94]; p=0·025; table 2). The intracluster correlation coefficient for the incidence rate of *P vivax* malaria over 6 months was 0·07 (95% CI 0·01–0·20). In the cluster-level analysis, the incidence rate of *P vivax* recurrence was 538 (95% CI 312–765) infections per 1000 person-years observed in the supervised group versus 862 (623–1100) in the unsupervised group (p=0·053; appendix 2 p 9). Overall, 73 (63%) of 116 *P vivax* or mixed recurrences and 67 (71%) of 95 *P falciparum* recurrences were symptomatic at the time of presentation to the clinic.

In the 195 patients who initially presented with *P vivax* infection (monoinfection or mixed species), the risk of the first *P vivax* recurrence at 6 months was significantly lower in the supervised group than in the unsupervised group (HR 0·15 [95% CI 0·02–0·95]; p=0·045; table 2). Although the incidence rate of all *P vivax* recurrences was also lower in the supervised group than in the unsupervised group, this finding was not significant (IRR 0·71 [95% CI 0·42–1·20]; p=0·20). In the 224 patients who initially presented with *P falciparum* infection, the corresponding HR at 6 months was 0·33 (95% CI 0·08–1·44; p=0·14) and the IRR was 0·52 (95% CI 0·28–0·98; p=0·043).

The cumulative incidence risk of any recurrent parasitaemia at 6 months was 50·0% (95% CI 35·1–67·1) in the supervised group versus 80·6% (49·9–98·0) in the unsupervised group (HR 0·17 [95% CI 0·06–0·45]; p=0·0005). In post-hoc analyses that examined the comparative risks of *P falciparum* recurrence, the cumulative incidence risk of *P falciparum* parasitaemia at 6 months was 27·1% (95% CI 21·2–34·2) in the supervised group and 36·4% (21·8–56·5) in the unsupervised group; the corresponding incidence rates for all *P falciparum* infections were 646 (95% CI 503–830) infections per 1000 person-years and 544 (405–730), respectively. Neither the incidence risk nor the rate of *P falciparum* recurrence differed significantly between treatment groups.

The total dose of primaquine administered was calculated in all 223 patients in the supervised group and 155 (79%) of 196 in the unsupervised group. Although the median total dose of primaquine prescribed was similar between groups (7·3 mg/kg [IQR 6·1–8·2] in the supervised group and 7·1 mg/kg [5·7–8·0] in the unsupervised group), only 126 (64%) patients in the unsupervised group took 5 mg/kg or greater, compared with 199 (89%) in the supervised group.

**Figure 3:**
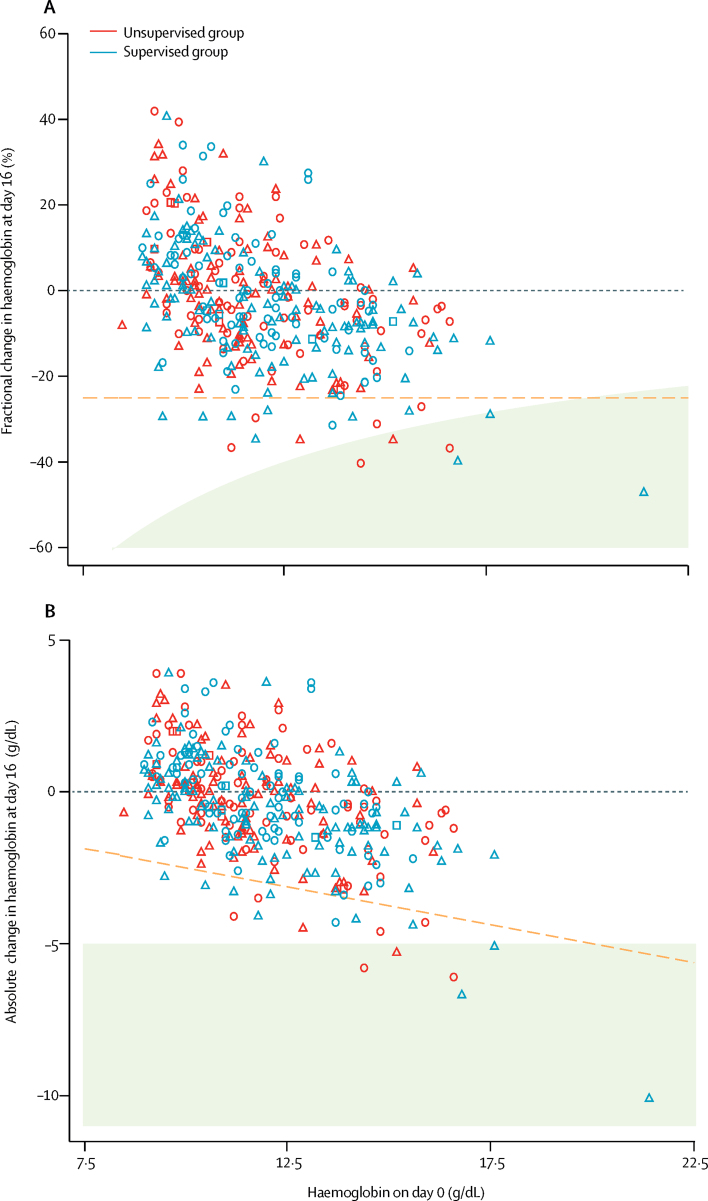
Relative and absolute change in haemoglobin from before (day 0) to after 14 days of primaquine treatment (day 16) (A) Relative change in haemoglobin. (B) Absolute change in haemoglobin. 171 participants in the unsupervised group (blue) and 195 in the supervised group (red) had measurements at both timepoints. Circles denote participants enrolled with *Plasmodium vivax*, triangles denote those enrolled with *Plasmodium falciparum*, and squares denote those enrolled with mixed *P vivax* and *P falciparum* infection. The dashed orange lines represent a fractional fall of 25%. The shaded area on both graphs represents an absolute fall of greater than 5 g/dL.

Overall, the mean haemoglobin concentration fell from 11·9 g/dL (95% CI 11·7–12·1) at baseline to 11·2 g/dL (11·1–11·4) on day 2, before the first dose of primaquine. The fractional fall in haemoglobin on day 2 was 5·9% (95% CI 4·3–7·5) in patients with *P falciparum* infection compared with 4·2% (2·4–5·9) in patients with *P vivax* infection (p=0·15). On day 16, after patients had completed primaquine treatment, the mean haemoglobin among patients in the supervised group was 11·5 g/dL (95% CI 11·3–11 ·7) and 11 (6%) of 195 patients had a fractional fall of more than 25% from baseline; the mean haemoglobin among patients in the unsupervised group was 11·6 g/dL (11·4–11·8) and eight (5%) of 171 patients had a fractional fall from of more than 25% from baseline (figure 3). Three patients in each treatment group had a fall in haemoglobin of more than 5 g/dL from baseline (four with *P falciparum* infection and two with *P vivax)*. One male patient aged 15 years, who presented with falciparum malaria and was treated with supervised primaquine, had a clinically significant fall in haemoglobin, from 9·5 g/dL at baseline to 6·7 g/dL on day 16; he remained asymptomatic and did not require hospitalisation, and by day 28 his haemoglobin had risen to 9·0 g/dL without the need for blood transfusion. The haematological profiles during follow-up by treatment group are presented in appendix 2 (p 10). After day 16, at least one episode of anaemia (haemoglobin <10 g/dL) had occurred in 77 (39%) of 196 patients in the unsupervised group, compared with 76 (34%) of 222 in the supervised group.

The analysis of early primaquine tolerability between days 2 and 16 was confined to patients in the supervised group; participants in the unsupervised group were assessed on days 2 and 16 only. None of the patients in the supervised group reported vomiting their primaquine dose within 1 h of administration. 23 (11%) of 215 patients in the supervised group had gastrointestinal symptoms during their course of primaquine treatment (days 2–16; table 3). Seven serious adverse events were reported (three in the supervised group, four in the unsupervised group), none of which were related to dihydroartemisinin–piperaquine or primaquine treatment. Four patients were admitted to hospital with complications of acute falciparum malaria either at the initial or subsequent presentations and three patients were hospitalised due to measles with bronchopneumonia, acute cholecystitis, and community acquired pneumonia respectively (appendix 2 p 11). No patients died or required blood transfusion.

**Table 3:** Safety endpoints

	**Supervised group (n=223)**	**Unsupervised group (n=196)**
**Within 1 h**		
Vomiting any dose of dihydroartemisinin–piperaquine	7/222 (3%)	11/190 (6%)
Vomiting any dose of primaquine	0/210	NA
**Days 2–16**		
Vomiting in previous 24 h	1/212 (<1%)*; 4/215 (2%)†	1/193 (1%)*
Headache	19/212 (9%)*; 36/215 (17%)†	21/193 (11%)*
Nausea	6/212 (3%)*; 6/215 (3%)†	7/193 (4%)*
Diarrhoea	1/212 (<1%)*; 4/215 (2%)†	0/193*
Skin rash or itching	0/212*; 1/215 (<1%)†	1/193 (1%)*
Poor appetite	3/212 (1%)*; 10/215 (5%)†	4/193 (2%)*
Abdominal pain	2/212 (1%)*; 6/215 (3%)†	1/193 (1%)*
Myalgia or arthralgia	12/212 (6%)*; 29/215 (13%)†	8/193 (4%)*
Fever	19/212 (9%)*; 35/215 (16%)†	13/193 (7%)*
Passing dark urine	1/212 (<1%)*; 2/215 (1%)†	0/193*
Dizziness	6/212 (3%)*; 16/215 (7%)†	13/193 (7%)*
Any gastrointestinal symptoms‡	11/212 (5%)*; 23/215 (11%)†	11/193 (6%)*
**Within 28 days**		
Serious adverse event related to primaquine§	0/222	0/196
Serious adverse event unrelated to primaquine	0/222	0/196
Within 6 months		
Serious adverse event related to primaquine§	0/222	0/196
Serious adverse events unrelated to primaquine	3/222 (1%)	4/196 (2%)

Data are n/N (%). Symptoms were elicited from daily questionnaires during treatment; participants in the unsupervised group were assessed on days 2 and 16 only, whereas those in the supervised group were assessed at each supervised visit (days 2, 4, 6, 8, 10, 12, 14, and 16). Serious adverse events include events of all severities. NA=not applicable.

* Proportion of patients reporting each symptom at least once on day 2 or day 16.

† Proportion of patients reporting each symptom at least once on any day between day 2 and day 16 inclusive.

‡ Composite of nausea, vomiting, anorexia, diarrhoea, or abdominal pain.

§ Related to primaquine includes possibly, probably, and definitely related.

## Discussion

This study shows that supervision of primaquine treatment on alternate days resulted in significantly better efficacy than the current practice of unsupervised primaquine, reducing the risk of any *P vivax* recurrence in 6 months by 77% and the rate of recurrences by 37%. The benefits of supervised primaquine radical cure were apparent in patients who presented with either *P vivax* or *P falciparum* malaria.

We have previously shown that 10–15% of patients who presented with either *P vivax* or *P falciparum* malaria and who were treated with dihydroartemisinin–piperaquine alone had recurrent *P vivax* infection within 42 days.^19^ In the current trial, the addition of primaquine reduced the risk of *P vivax* recurrence almost three-fold at a similar timepoint and this finding was apparent in patients treated with either supervised or unsupervised primaquine. In a large population study in southern Papua, the effectiveness of unsupervised primaquine in routine clinical practice was estimated to be only 12%,^7^ whereas the effectiveness of unsupervised primaquine in our current study was considerably higher. It is possible that selection and consent of patients into a formal clinical trial and supervision of the initial 3 days of schizontocidal therapy might have enhanced patient adherence to a full treatment course, despite minimal supervision of treatment thereafter. Therefore, supervision of the first dose of primaquine and provision of education might provide a pragmatic approach to improving treatment adherence.

In the current study, patient follow-up continued for 6 months and each recurrence was treated with the same regimen as assigned at randomisation. At the end of follow-up, there were significantly more *P vivax* recurrences in those who received unsupervised primaquine treatment than in those who received supervised primaquine treatment (859 infections per 1000 patient years *vs* 539; p=0·025), although the difference didn't begin to emerge until after 90 days (figure 2). The prolonged post-exposure prophylaxis provided by piperaquine for the initial blood stage treatment might have suppressed or delayed relapses and masked early differences in anti-relapse efficacy between treatment groups.^19^ For individuals residing in an endemic setting, recurrent malaria can be due to recrudescence (schizontocidal failure), reinfection from a new mosquito bite, or relapse from hypnozoite reactivation. Although we were unable to distinguish between these scenarios, recrudescent infections are likely to be only a minor factor, because they usually occur within 63 days, and efficacy trials and genomic analyses have found no evidence of parasite resistance to either artemisinin or piperaquine.^20^ As the clusters were matched according to the level of malaria transmission, the risk of reinfection is likely to have been similar between treatment groups. Pooled analyses of longitudinal cohorts have shown that relapses account for more than 60–90% of recurrences across a range of endemic settings.^21^ Hence, the most likely explanation is higher anti-relapse efficacy in the supervised group, and this is supported by the higher proportion of patients taking a total dose of primaquine of at least 5 mg/kg than in the unsupervised group.

A key finding of our study was the significant benefit of providing effective radical cure to patients presenting with *P falciparum* monoinfection. Antimalarial guidelines currently restrict the use of primaquine in patients presenting with acute *P falciparum* to a single dose of 0·25 mg/kg for its gametocytocidal activity and reduction of transmission; however, this regimen does not kill *P vivax* hypnozoites. In the current study, patients presenting with *P falciparum* received the same treatment as those presenting with *P vivax*, a 14-day high-dose regimen. By 6 months, the risk of *P vivax* recurrence following initial *P falciparum* infection was 56% in the unsupervised group and 31% in the supervised group, equating to a 67% reduction in the risk of *P vivax* and a 48% reduction in the rate of recurrences. In the study region, the incidence of malaria is approximately 250 cases per 1000 population per year for both *P falciparum* and *P vivax*, and recurrent episodes of vivax and falciparum malaria are common, which suggests that there is a high burden of latent hypnozoite carriage in all patients presenting with malaria.^7^, ^14^ Our study provides further evidence that opportunistically targeting patients with *P falciparum* infection, who are at high risk of carrying occult hypnozoites, can reduce recurrent clinical illness and potentially reduce ongoing transmission.^22^ The benefits of primaquine radical cure for patients with *P falciparum* that we observed are likely to be conservative, as they represent comparison of complete with partial adherence to 14-day primaquine, rather than comparison with the current practice of single-dose primaquine. The rationale for the broader use of hypnozoitocidal drugs is likely to apply similarly for tafenoquine, a slowly eliminated 8-aminoquinoline drug, that has been licensed as a single-dose regimen for *P vivax* radical cure.^23^ Although tafenoquine has advantages over primaquine, and avoids the challenges of adherence, it is currently only recommended for use with chloroquine. Such a policy precludes its use in countries such as Indonesia, where a universal policy of artemisinin-based combination therapy has been adopted for uncomplicated malaria due to any *Plasmodium* species of malaria.

In patients with normal G6PD levels (>30% activity), the high-dose 14-day primaquine regimen was well tolerated and was not associated with severe adverse complications. However, six patients had a fall in haemoglobin greater than 5 g/dL, four of whom presented with *P falciparum* infection (figure 3), which might have been exacerbated by concomitant primaquine administration. These events occurred in patients who presented with haemoglobin greater than 14 g/dL, none of whom became clinically unwell, and their haemoglobin concentrations remained normal until the end of follow-up. Acute malaria results in inevitable parasite-induced haemolysis and this is usually greater following *P falciparum* infection than with *P vivax* infection.^24^, ^25^ Previous pooled analyses have shown that although primaquine can cause an initial excess reduction in haemoglobin, recovery is usually rapid and often offset by a reduction in subsequent recurrent parasitaemia and further parasite-induced haemolysis.^24^ In the current study, fewer people in the supervised primaquine group developed anaemia during follow-up than in the unsupervised group.

Gastrointestinal intolerance is an acknowledged side-effect of primaquine and although it is dose-related, it can be reduced by concomitant administration of food. Less than 5% of patients vomited their dose of primaquine within 1 h of administration, and subsequent rates of vomiting after clinical recovery were even lower. Two patients with normal G6PD levels reported dark urine, but subsequent investigation suggested that the dark colour was due to dehydration rather than haemoglobinuria. None of the patients required admission to hospital due to primaquine treatment (appendix 2 p 11).

The risk and frequency of *P vivax* relapses, and the corresponding dose of primaquine required to achieve high anti-relapse efficacy, vary considerably between geographical regions.^2^, ^3^ When fully supervised, a high-dose primaquine regimen (total dose 7 mg/kg, similar to that used in our study) has been shown to result in a low risk of *P vivax* relapse, with the risk at 12 months ranging from 7% to 20%,^26^ compared with 31–41% for patients treated with low-dose primaquine or tafenoquine.^23^, ^27^, ^28^ The risk of *P vivax* following *P falciparum* also varies with the background prevalence of malaria and relapse periodicity.^2^, ^12^ Such diversity will influence the risks, benefits, and cost-effectiveness of supervising primaquine radical cure and broadening its use to include patients with *P falciparum* malaria. Further studies addressing these issues in different endemic settings are warranted.

Our study has some limitations. Follow-up was incomplete, with only 72–77% of participants contributing to the primary endpoint. The loss to follow-up was attributable to the high patient mobility of migrant workers. Loss to follow-up might have selected patients with a more positive attitude towards treatment adherence and thus led to underestimation of the true effect of the intervention. In the unsupervised treatment clusters, the study provided more consultation time with clinic or study staff than patients would have in real-world settings,^7^ and this is likely to have resulted in a Hawthorne effect—modifying patients' behaviour and leading to higher than expected treatment adherence,^29^, ^30^ both of which will also have led to underestimation of the effect of the intervention. A further limitation was the significantly higher risk of *P falciparum* infections in the supervised group than in the unsupervised group, potentially reflecting greater heterogeneity between clusters than expected. A higher intracluster correlation coefficient than predicted might have led to the study being underpowered, particularly for the secondary analyses.

In conclusion, partial supervision of 14-day primaquine treatment enhanced adherence and reduced incidence and rates of recurrent *P vivax* infection significantly, in patients presenting with either *P vivax* or *P falciparum* malaria. Extending the use of safe and effective primaquine radical cure to patients presenting with non-vivax malaria should be considered, but will need to be tailored to areas with a high risk of *P viva*x recurrence after *P falciparum* infection.^12^ As supervision on alternate days might not be feasible in routine clinical practice, particularly in areas with high case numbers, more parsimonious strategies should be explored, combining patient education and early clinical review to promote adherence or curtail treatment if signs of an impending haemolysis occur. These strategies could have particular relevance in implementing high-dose 7-day primaquine regimens.^26^ Greater access to safe and effective radical cure has potential to reduce the burden of *P vivax* substantially, paving the way for ambitious malaria elimination targets to be met.

## Data sharing

The data are available for access via the WorldWide Antimalarial Resistance Network (WWARN). Requests for access will be reviewed by a data access committee to ensure that use of data protects the interests of the participants and researchers according to the terms of ethics approval and principles of equitable data sharing. The study protocol and statistical analysis plan are provided in the appendix. Access to individual deidentified patient data will be made available following publication. Requests can be submitted by email to malariaDAC@iddo.org via the data access form available at https://www.wwarn.org/working-together/sharing-accessing-data/accessing-data. The WWARN is registered with the Registry of Research Data Repositories.

## Declaration of interests

We declare no competing interests.

## Supplementary Materials

Indonesian translation of the abstract
