# Supplementary material for: Supervised versus unsupervised primaquine radical cure for the treatment of falciparum and vivax malaria in Papua, Indonesia: a cluster-randomised, controlled, open-label superiority trial
Source: Lancet Infect Dis. 2022 Mar;22(3):367–76. doi: 10.1016/S1473-3099(21)00358-3 (PMC8866132; doi:10.1016/S1473-3099(21)00358-3)
Supplement: Indonesian translation of the abstract [file mmc1.pdf]

THE LANCET
Infectious Diseases

Supplementary appendix 1

This translation in Indonesian was submitted by the authors and we reproduce it as supplied. It has not been peer reviewed. The Lancet's editorial processes have only been applied to the original in English, which should serve as reference for this manuscript.

Supplement to: Poespoprodjo JR, Burdam FH, Candrawati F, et al. Supervised versus unsupervised primaquine radical cure for the treatment of falciparum and vivax malaria in Papua, Indonesia: a cluster-randomised, controlled, open-label superiority trial. *Lancet Infect Dis* 2021; published online oct Oct 25. https://doi.org/10.1016/S1473-3099(21)00358-3.

Terjemahan dalam bahasa Indonesia ini diserahkan oleh penulis dan kami memperbanyaknya sebagaimana disediakan. Itu belum ditinjau oleh rekan sejawat. Proses editorial Lancet hanya diterapkan pada aslinya dalam bahasa Inggris, yang seharusnya menjadi referensi untuk manuskrip ini.

Pengobatan radikal dengan primakuin dengan pengawasan vs tanpa pengawasan minum obat sebagai terapi malaria falsiparum dan vivax di Papua, Indonesia: Penelitian superioritas klaster acak, terkontrol, label terbuka.

Latar belakang. Risiko terjadinya rekurensi *Plasmodium vivax* pada pasien dengan *P. falciparum* di daerah ko-endemik malaria cukup tinggi. Terapi radikal dengan primakuin mempunyai potensi untuk dapat menurunkan kejadian rekurensi *P. vivax* pada pasien dengan malaria falsiparum maupun vivax, namun pada prakteknya sulit dilakukan karena rendahnya ketaatan minum obat selama 14 hari. Penelitian ini bertujuan untuk menilai efikasi dan keamanan pemberian primakuin sebagai terapi radikal dengan pengawasan dan tanpa pengawasan minum obat pada pasien dengan malaria tanpa komplikasi.

Metoda. Kami melakukan penelitian superioritas klaster acak, terkontrol, label terbuka di Papua, Indonesia. Sebanyak 21 klaster pos kesehatan desa, yang di cocokan berdasarkan *annual parasite index*, kami acak (1:1) untuk menerima perlakuan pemberian dihidroartemisinin-piperakuin oral dengan primakuin 14 hari (0,5 mg/kg/hari) dengan pengawasan atau tanpa pengawasan pada pasien (usia>12 bulan dan berat badan >5kg) dengan malaria falsiparum atau vivax tanpa komplikasi. Pasien pada kelompok pengawasan, pengobatan primakuin diawasi setiap 2 hari. Pasien diikuti selama 6 bulan dan pasien yang mengalami malaria berulang diterapi dengan protokol pengobatan yang sama. Pembutaan perlakuan tidak memungkinkan karena kondisi penelitian. Luaran primer adalah *incidence risk* kejadian *P. vivax* dalam kurun waktu 6 bulan, yang dinilai pada *modified intention to treat population* (semua pasien yang dialokasikan ke kelompok pengobatan, tanpa memasukan pasien yang tidak datang kembali setelah kunjungan pertama). Penelitian ini telah selesai dan terdaftar pada ClinicalTrials.gov, NCT02787070.

Hasil. Mulai tanggal 14 September 2016 sampai dengan 31 Juli 2018, sebanyak 436 pasien dilakukan penapisan untuk ikut dalam penelitian dan 419 pasien memenuhi kriteria inklusi; 223 (53%) pasien dari 11 klaster di berikan terapi primakuin dengan pengawasan dan 196 (47%) pasien pada 10 klaster diberikan terapi primakuin tanpa pengawasan. 161 (72%) dari 223 pasien di kelompok pengawasan dan 151 (77%) dari 196 di kelompok tanpa pengawasan dapat mengikuti *follow up* selama 6 bulan. Pada bulan ke 6, *incidence risk* rekurensi *P. vivax* pada kelompok dengan pengawasan adalah 29,7% (95% CI 16,4–49,9) versus 55,8% (32,3–81,8) pada kelompok tanpa pengawasan (*hazard ratio* 0,23 [95% CI 0,07–0,76]; p=0,016). *Incidence rate* rekurensi *P. vivax* adalah 539 (95% CI 390–747) infeksi per 1000 orang-tahun pada kelompok dengan pengawasan versus 859 (673–1096) pada kelompok yang tanpa pengawasan (*incidence rate ratio* 0,63 [95% CI 0,42–0,94]; p=0,025). *Incidence rate* dengan urutan yang sama pada 224 pasien dengan malaria falsiparum adalah 660 (95% CI 446–977) dan 346 (213–563; *incidence rate ratio* 0,52 [95% CI 0,28–0,98]; p=0,043). Dilaporkan terdapat 7 kejadian buruk berat (tiga pada kelompok dengan pengawasan, empat pada kelompok yang tanpa pengawasan) dan tidak ada satupun yang berhubungan dengan intervensi yang diberikan, dan tidak didapatkan kasus kematian.

Interpretasi. Pada daerah dengan transmisi malaria sedang, pemberian terapi primakuin dengan pengawasan mengurangi risiko rekurensi *P. vivax*. Hasil ini tampak jelas baik pada pasien malaria falsiparum maupun vivax. Penelitian selanjutnya perlu dilakukan untuk melihat keamanan dan efikasi terapi radikal pada pasien dengan malaria falsiparum tanpa komplikasi di daerah ko-endemik lain.
