# Supplementary material for: Supervised versus unsupervised primaquine radical cure for the treatment of falciparum and vivax malaria in Papua, Indonesia: a cluster-randomised, controlled, open-label superiority trial
Source: Lancet Infect Dis. 2022 Mar;22(3):367–76. doi: 10.1016/S1473-3099(21)00358-3 (PMC8866132; doi:10.1016/S1473-3099(21)00358-3)
Supplement: Supplementary appendix 2 [file mmc2.pdf]

THE LANCET
Infectious Diseases

Supplementary appendix 2

This appendix formed part of the original submission and has been peer reviewed. We post it as supplied by the authors.

Supplement to: Poespoprodjo JR, Burdam FH, Candrawati F, et al. Supervised versus unsupervised primaquine radical cure for the treatment of falciparum and vivax malaria in Papua, Indonesia: a cluster-randomised, controlled, open-label superiority trial. *Lancet Infect Dis* 2021; published online oct Oct 25. https://doi.org/10.1016/S1473-3099(21)00358-3.

Supplementary Appendices

Supplement to: **Supervised versus unsupervised primaquine radical cure for the treatment of falciparum and vivax malaria in Papua, Indonesia: a cluster randomised, controlled, open-label superiority trial**

Table of contents

1. Public clinic malaria cases reported in 2017 2

2. Dosing Regimen 3

3. Statistical Analysis Plan 4-7

4. Histogram of the number of Plasmodium vivax and falciparum recurrences 8

5. Incidence Rates of P. vivax for each cluster..9

6. Distribution of haemoglobin (g/dl) during follow up by treatment arm 10

7. Details of Severe Adverse Events (SAEs) 11

8. TRIPI Protocol vs 1.1 12

1. Public clinic malaria case numbers reported in 2017

Primary Healthcare Clinics	Cluster	Village Health Post	Group	Population	Malaria cases	API
**Limau Asri**	9	Mulia Kencana (SP7)	Supervised	2,064	44	21.32
**Limau Asri**	10	Naena Muktipura (SP6)	Unsupervised	1,688	37	21.92
**Mapuru Jaya**	15	Pigapu (Logpong)	Supervised	435	9	20.69
**Pasar Sentral**	5	Sempan (Inauga)	Unsupervised	11,715	262	22.36
**Kwamki**	7	Harapan Baru	Supervised	8,654	200	23.11
**Wania**	1	Kamoro Jaya (SP1)	Unsupervised	15,776	364	23.07
**Timika**	6	Wonosari Jaya (SP4)	Supervised	8,710	201	23.08
**Mapuru Jaya**	17	Tipuka	Unsupervised	564	13	23.05
**Timika**	4	Nawaripi	Supervised	5,743	133	23.16
**Mapuru Jaya**	13	Kadun Jaya	Unsupervised	3,411	111	32.54
**Limau Asri**	11	Wangirja (SP9)	Supervised	1,687	40	23.71
**Pasar Sentral**	3	Kwamki Baru	Unsupervised	20,849	733	35.16
**Mapuru Jaya**	18	Wania village	Supervised	1,296	43	33.18
**Mapuru Jaya**	8	Mware	Unsupervised	1,292	52	40.25
**Mapuru Jaya**	14	Kaugapu	Supervised	1,597	55	34.44
**Limau Asri**	21	Utikini Baru (SP12)	Unsupervised	4,709	192	40.77
**Jilli Yalle**	20	Karang Senang (SP3)	Supervised	9,242	328	35.49
**Mapuru Jaya**	16	Poumako	Unsupervised	3,207	192	59.87
**Bhintuka/SP13**	19	Bhintuka (SP13)	Supervised	2,080	89	42.79
**Mapuru Jaya**	12	Hiripau	Unsupervised	1,751	127	72.53
**Timika**	2	Koperapoka	Supervised	18,261	447	24.48

API – Annual Parasite Index per 1000 individuals

2. Dosing Regimen

2.1 Schizontocidal Treatment

Each tablet contains 40 mg dihydroartemisinin (DHA) and 320 mg piperaquine

Body weight (kg)	Number of tablets			Total Target Dose (mg/kg)	
	Day 0	Day 1	Day 2	DHA	Piperaquine
=5	1/4	1/4	1/4	6.0	48
6 - 10	1/2	1/2	1/2	6.0 - 10	48 - 80
11 - 17	1	1	1	7.1 - 10.9	57 - 87
18 - 30	1 ½	1 ½	1 ½	6.0 - 10	48 - 80
31 - 40	2	2	2	6.0 - 7.7	48 - 62
41 - 60	3	3	3	6.0 - 8.8	48 - 70
61 - 80	4	4	4	6.0 - 7.9	48 - 63
81 - 100	5	5	5	6.0 - 7.4	48 - 59

Rescue medication. Patients failing to respond to the trial drug were prescribed unsupervised quinine plus clindamycin for 7 days (Dose: quinine 10 mg/kg body weight 3 times/day and clindamycin 5 mg/kg body weight 3 times/day). Parasitological and clinical response were reviewed at day 3 and 7 after commencing oral quinine therapy.

If manifestations of severe malaria occurred, intravenous artesunate (2.4 mg/kg bodyweight at 0, 12 and 24 hours) or intravenous quinine (10 mg/kg bodyweight 8 hourly) was administered immediately along with the required supportive treatments according to the hospital protocol.

2.2 Hypnozoitocidal treatment

Primaquine (PQ) (each tablet contains 15 mg primaquine) was administered daily for 14 days with food (daily target dose 0.5mg / kg BW (body weight)):

Weight (kg)	Number of tablets per day for 14 days	Daily dose of PQ (mg / kg BW)	Total dose of PQ (mg / kg BW)
5.0 - 24.9	0.5	0.3 - 1.5	4.2 - 21
25.0 - 34.9	1	0.4 - 0.6	6.0 - 8.4
35.0 - 45.9	1.5	0.5 - 0.6	6.9 - 9.0
≥ 46.0	2	≤0.7	≤ 9.1

3. Statistical Analysis Plan

A. Introduction and Rationale

This study is a health care facility based, cluster randomised, controlled, open label trial comparing supervised versus unsupervised primaquine (PQ) treatment to prevent recurrences of *Plasmodium vivax* parasitemia in patients with acute, uncomplicated malaria infection due to either *P. falciparum* or *P. vivax*.

P. vivax can form dormant liver stages (hypnozoites) that reactivate weeks or months following an acute infection. Recurrent infections can be associated with a febrile illness, a cumulative risk of severe anaemia, and mortality. In co-endemic areas, the risk of recurrence after both *P. vivax* and *P. falciparum* infections can be over 50% within 3 months. The only drug available to target *P. vivax* hypnozoites is PQ, which is currently given as a 14-day regimen. In Papua, a retrospective study found very low effectiveness for unsupervised treatment. If true, this has profound effects on treatment policy, suggesting that greater efforts are needed to encourage adherence to treatment.

Our cluster randomised, controlled, open-label trial assessed the effectiveness of unsupervised versus supervised PQ treatment in patients with uncomplicated malaria. Since the risk of recurrent *P. vivax* is high in patients with either *P. vivax* or *P. falciparum,* both infections were included in the study. The study was conducted in southern Papua Province, Indonesia. Participants were enrolled at village health posts and provided with schizontocidal treatment plus PQ radical cure, which was either supervised or unsupervised depending on which cluster the clinic was located. Participants were followed for 6 months and assessed at regular intervals for the presence of patent and sub-patent malaria.

The ultimate goal of the study was to quantify the adherence to radical cure treatment and its associated impact on the risk of recurrence, so that public health resources can be focused on developing novel approaches to improve effectiveness.

The SAP is based on the version of the amended protocol (V1.1 29 May 2017) and approved by the Research Ethics Committees of the University of Gadjah Mada, Indonesia and Menzies School of Health Research, Darwin, Australia.

Aim of the study
To assess the effectiveness of radical cure in G6PD normal patients with uncomplicated malaria on reducing recurrent parasitaemia.

Eligibility criteria
Essential inclusion criteria

- Infection with *P. falciparum* or *P. vivax*, either alone or mixed
- Age >12 months
- Weight >5kg
- Living in the study clusters

Exclusion Criteria

- General danger signs or symptoms of severe malaria
- Anaemia, defined as haemoglobin (Hb) level <9g/dl
- G6PD deficiency (as determined by fluorescent blood spot test (FST))
- Pregnant women as determined by urine β-HCG pregnancy test
- Known hypersensitivity to any of the study drugs

Randomised Study Arms

A total of 21 clusters were recruited, and each cluster recruited 20 participants, giving a total of 419 participants enrolled in the study. All participants received dihydroartemisinin (DHP) 2-4mg/kg body weight and piperaquine 16-32mg/kg body weight according to guidelines. The first three days of DHP were supervised in all patients independent of the cluster. If the participant vomited the treatment within 60 minutes of dosage a repeat dose was administered.

The patients were reviewed on day 2 after the third dose of DHP. If haemoglobin levels were above 9g/dL, the patient was given PQ (0.5mg/kg/day) for 14 days as per local guidelines for radical cure. According to the randomisation cluster, the PQ regimen was either unsupervised (current standard practice) or supervised on alternate days.

The same treatment allocation was applied for every symptomatic and asymptomatic malaria episode occurring during the follow up. During subsequent malaria episodes radical cure (PQ) was provided together with schizontocidal treatment (DHP). Schizontocidal treatment on day 2 and 3 was unsupervised in the non-supervised arm during subsequent malaria episodes after enrolment.

Data Collection

The data were collected on paper clinical record forms (CRF) which were then digitalised into a custom-made Epidata DataBase (Denmark).

B. Objectives, Endpoints and Definitions

Specific objectives and endpoints of the study

Primary Objective:

To assess the effectiveness of unsupervised compared to supervised 14-day PQ treatment regimens in preventing recurrent *P. vivax* parasitaemia in G6PD normal patients presenting with uncomplicated malaria.

Study endpoint(s):

- The incidence risk of the first recurrent episode of microscopic (symptomatic or asymptomatic) *P. vivax* malaria over 6 months in patients enrolled with uncomplicated *P. vivax* or *P. falciparum* malaria. (Primary Endpoint)

- The incidence rate of microscopic (symptomatic or asymptomatic) *P. vivax* malaria over 6 months in patients enrolled with uncomplicated *P. vivax* or *P. falciparum* malaria. (Secondary Endpoint)

Secondary Objectives:
To assess the effectiveness of unsupervised compared to supervised 14-day PQ treatment regimens in preventing recurrent *P. vivax* parasitaemia in G6PD normal patients presenting with uncomplicated *P. vivax* mono-infection.

Study endpoint(s):
- The incidence risk of the first recurrent episode of microscopic (symptomatic or asymptomatic) *P. vivax* malaria over 6 months in patients enrolled with uncomplicated *P. vivax* mono-infection.
- The incidence rate of microscopic (symptomatic or asymptomatic) *P. vivax* malaria over 6 months in patients enrolled with uncomplicated *P. vivax* mono-infection.

To assess the effectiveness of unsupervised compared to supervised 14-day PQ treatment regimens in preventing recurrent *P. vivax* parasitaemia in G6PD normal patients presenting with uncomplicated *P. falciparum* mono-infection.

Study endpoint(s):
- The incidence risk of the first recurrent episode of microscopic (symptomatic or asymptomatic) *P. vivax* malaria over 6 months in patients enrolled with uncomplicated *P. falciparum* mono-infection.
- The incidence rate of microscopic (symptomatic or asymptomatic) *P. vivax* malaria over 6 months in patients enrolled with uncomplicated *P. falciparum* mono-infection.

To quantify the comparative tolerability of supervised and unsupervised PQ.

Study endpoint(s):
- The proportion of patients vomiting their medication within 1 hour of administration.
- The proportion of patients vomiting any of their PQ doses during the 14-day supervised course.
- The proportion of patients with adverse symptoms from day 2 to 16, including headache, muscle or joint pain, abdominal pain, poor appetite, nausea, vomiting, diarrhoea, dark urine, skin rash or itching, dizziness, and other symptoms.
- The proportion of adverse events and serious adverse events over 6 months in all patients.
- The incidence risk of severe anaemia (Hb <7g/dl) and/or the risk for blood transfusion over 6 months.
- The incidence risk of an acute drop in haemoglobin (a decrease in Hb >5g/dl compared to baseline Hb) or fractional fall of >25% in Hb to Hb less than 7 g/dl within 14 days of starting PQ treatment.

Definitions of Endpoints

- **Incidence risk** refers to the conditional probability of the first occurrence of the event of interest (e.g. symptomatic *P. vivax* recurrence) within a specified timeframe (e.g. within 6 months of presentation). Incidence risk is conditional on that individual having not yet experienced the event, and so patients are censored from when the event occurs, or from time of last follow up if the event does not occur.
- **Incidence rate** is the rate at which the event of interest occurs and is expressed as expected events per a given unit of time. This analysis accounts for multiple occurrences of the event of interest over the follow up period. Incidence rate is calculated using the number of events divided by the total number of days of follow up.

Patient, treatment and disease characteristics and definitions
The following characteristics will be examined:

- **Patient:** age, sex, weight, history of fever in the last 48 hours, fever (>37.5^{0}C axillary), ethnicity, history of malaria in the last 28 days
- **Treatment**: schizontocidal treatment (mg/kg dose), hypnozontocial treatment (mg/kg dose), supervision of schizontocidal treatment, adherence to PQ treatment (fullly adherent or non-adherent), pill count (unsupervised group only)
- **Laboratory:** parasitaemia, gametocytaemia, haemoglobin concentration

Age will be stratified into 1 to <5 years, 5 to <15 years, and 15 years or older. Ethnicity will be categorised as: Highland Papuan, Lowland Papuan or Non-Papuan. Schizontocidal treatment (DHP) will be classified as supervised if all doses were directly observed, and not-supervised if fewer doses were observed. PQ treatment in the supervised arm will be classified as fully adherent if doses on the alternate supervised days were all recorded as having been taken and the patient reports adherence on alternate days when they self-medicated. Otherwise they are classified as non-adherent. PQ treatment in the unsupervised arm will be classified as adherent if the patient reports adherence on each day and this is confirmed by the pill count on day 16. The doses of treatment received (i.e. PQ and DHP) will be calculated from the number of daily tablets administered to each patient. For each component, a total dose per weight will be calculated for each patient.

4. Histogram of the number of *Plasmodium vivax* recurrences (red columns) and *P. falciparum* recurrences (blue columns) during 180 days of follow up, stratified by (A) treatment arm and (B) species of infection at enrolment.

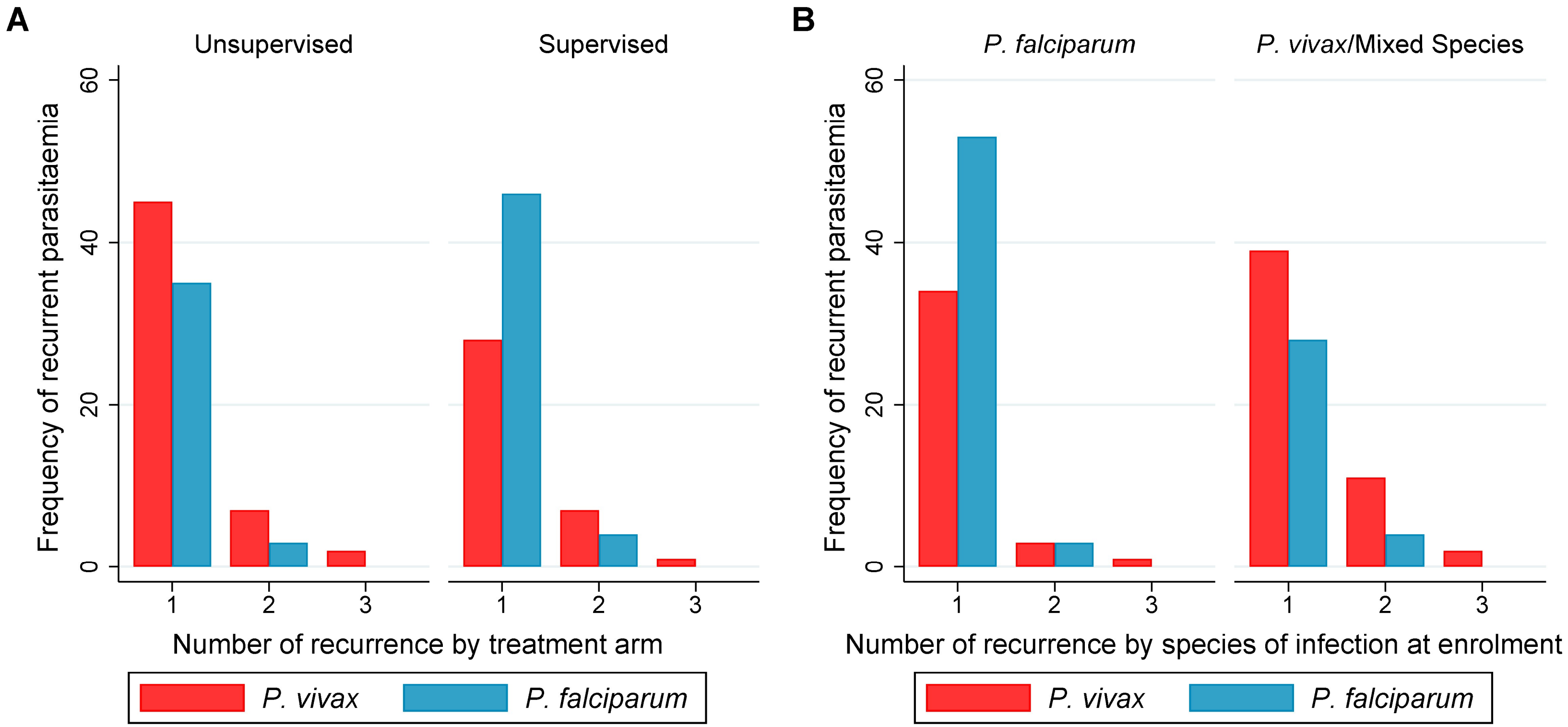

5. Incidence Rates of *P. vivax* for each cluster

Cluster	Village Health Post	Group			Observed Incidence Rate of *P. vivax* Per 1000-person years		
			**Number of PV Episodes**	**Years of Observation**	**Any malaria at presentation**	**Patients presenting with *P. vivax* (mono or mixed infections)**	**Patients presenting with *P. falciparum* (mono-infections)**
9	Mulia Kencana (SP7)	Supervised	10	8.15	1227	2131	617
15	Pigapu (Logpong)	Supervised	2	7.84	255	0	308
7	Harapan Baru	Supervised	5	6.25	800	1798	436
6	Wonosari Jaya (SP4)	Supervised	1	8.51	118	0	182
4	Nawaripi	Supervised	3	8.58	350	554	201
11	Wangirja (SP9)	Supervised	2	8.09	247	432	0
2	Koperapoka	Supervised	2	4.16	481	853	0
18	Wania village	Supervised	6	8.43	712	376	1283
14	Kaugapu	Supervised	6	8.88	676	1016	405
20	Karang Senang (SP3)	Supervised	7	7.22	969	1409	337
19	Bhintuka (SP13)	Supervised	1	7.51	133	252	0
		Overall	45	83.62	538	776	346
10	Naena Muktipura (SP6)	Unsupervised	9	8.13	1108	1547	817
5	Sempan (Inauga)	Unsupervised	3	5.54	542	1232	0
1	Kamoro Jaya (SP1)	Unsupervised	4	7.47	535	0	806
17	Tipuka	Unsupervised	5	6.48	772	1139	337
13	Kadun Jaya	Unsupervised	7	8.53	820	978	585
3	Kwamki Baru	Unsupervised	9	8.07	1115	755	1803
8	Mware	Unsupervised	10	7.93	1260	1436	980
21	Utikini Baru (SP12)	Unsupervised	5	7.34	681	826	653
16	Poumako	Unsupervised	11	8.48	1297	1876	842
12	Hiripau	Unsupervised	2	7.47	268	761	0
		Overall	65	75.43	862	1100	660

6. Distribution of haemoglobin (g/dl) during follow up by treatment arm

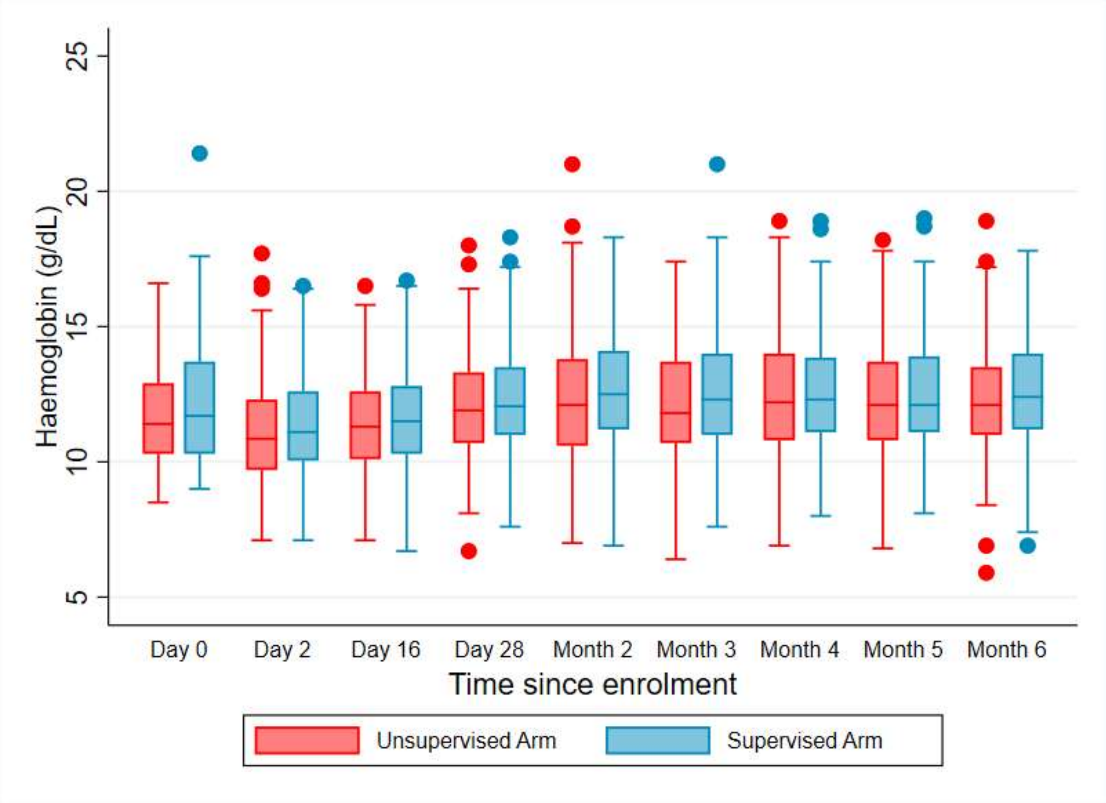

Boxes represent the 25th and 75th percentiles.

7. Details of Severe Adverse Events (SAEs)

No	Study ID	Intervention Arm	Sex	Age (years)	Date of enrolment	Clinical Assessment	Day of hospital admission	Days in hospital	Outcome Notes	Assessment of causality to study drug and study intervention
1	1.013	PQUnsup	M	56	23 Nov 2016	Acute falciparum malaria with cerebral complications	Day 1	3	Recovered	Unrelated
2	12.004	PQUnsup	M	11	27 Feb 2017	Acute falciparum malaria with intractable vomiting	Day 1	2	Recovered	Unrelated
3	15.014	PQSup	F	31	26 Sept 2016	Acute falciparum malaria with onset of jaundice prior to starting primaquine	Day 109	4	Recovered	Unrelated
4	21.003	PQUnsup	F	2	27 Sept 2016	Acute falciparum malaria with electrolyte imbalance	Day 139	4	Recovered	Unrelated
5	10.008	PQUnsup	M	1	12 Apr 2017	Measles with bronchopneumonia	Day39	9	Recovered	Unrelated
6	11.007	PQSup	M	28	31 Jan 2017	Acute cholecystitis	Day 99	3	Recovered	Unrelated
7	09.016	PQSup	F	23	29 Apr 2017	Community associated pneumonia	Day 93	5	Recovered	Unrelated

A randomized controlled trial on malaria primaquine treatment in Timika, Indonesia (TRIPI)

Version 1.1

Principal Investigators

Dr. Jeanne Rini Poespoprodjo, PhD
Timika Research Facility, Yayasan Pengembangan Kesehatan dan Masyarakat Papua, Timika, Papua, Indonesia
Paediatric Research Office, Department of Child Health, Universitas Gadjah Mada
Phone: +6281348430617
Email: didot2266@yahoo.com

Dr. Ratni Indrawanti, Sp.A
Department of Child Health
Faculty of Medicine, Universitas Gadjah Mada
Jl. Kesehatan no. 1, Sekip, Yogyakarta – 55284
Phone: +62 (274) 555 455
Email: Ratni_iw@yahoo.com

Professor Ric Price, MD
Menzies School of Health Research
PO Box 41096
Casuarina NT 0811
Australia
Phone: +618 89228197
Email: rprice@menzies.edu.au

Key Investigators:

Dr. Enny Kenangalem MBioMed, Timika Research Facility, Yayasan Pengembangan Kesehatan dan Masyarakat Papua, Timika, Papua, Indonesia

Dr Kamala Thriemer, Menzies School of Health Research, Darwin, Australia

Dr Benedikt Ley, Menzies School of Health Research, Darwin, Australia

Dr. Jutta Marfurt, Menzies School of Health Research, Darwin, Australia

Dr. Michelle Boyle, Menzies School of Health Research, Darwin, Australia

Other Collaborators:

Dr Rintis Noviyanti (Eijkmann Institute)
Molecular biology of drug resistance and red cell polymorphisms, genotyping, placental studies, spleen studies
Dr Julie Simpson (University of Melbourne, Australia)
Biostatistician. Advising on times series analysis.
Dr Freya Fowkes (Burnet Institute, Melbourne, Australia)
Scientist. Advising on serological studies.

Amendment history

Amendment number	Protocol version	Date	Submitted to	Changes
Original protocol	V 0.7	11.11.2015	HREC	
1	V 0.8		HREC	Study treatment also for asymptomatic episodes
2	V 0.9	25.01.2016	HREC	PI & Key investigators amended
3	V 1.1	12.01.2017	HREC	-Observational cohort added<br>-G6PD test algorithm modified<br>-Qualitative component added

Table of Contents

1 Executive Summary 5
2 Background and rationale 5
3 Aim 8
4 Study Objectives 8
5 Study design 8
5.1 Design and Rationale 8
5.2 Study site 9
5.3 Study population 9
5.4 Study duration and follow up period 9
5.5 Cluster Randomization 9
6 Treatment 10
6.1 Study treatment 10
6.1.1 Randomized arms 10
6.1.2 non-randomized cohort 10
6.2 Rescue treatment 11
6.3 Concomitant treatment 11
7 Socio-economic data collection 11
7.1 Quantitative Questionnaire 12
7.2. Qualitative interviews 12
8 Study procedures 13
8.1 Study procedures for the randomized arms 13
The study procedures are summarised in Table 1 13
8.1.1 Enrolment 13
8.1.2 Day 0 14
8.1.3 Day 1 14
8.1.4 Day 2 14
8.1.5 Days 3-15 (only participants in the supervised arm) 15
8.1.6 Day 16 15
8.1.7 Months 1-6 15
8.1.8 Day of recurrence 15
8.2 Procedures for serious adverse events 16
8.3 Discontinuation/ Withdrawal of Participants from Study Treatment 16
9 Study procedures for the non-randomized observational cohort 17
9.1 Inclusion Criteria 17
9.2 Exclusion Criteria 17
10 Laboratory procedures 20
10.1 Urine β-HCG pregnancy test 20
10.2 Blood collection 20
10.3 G6PD deficiency testing 20
10.4 Malaria Microscopy 20
10.5 Haemoglobin 21
10.6 Parasite molecular analysis 21
10.7 Host genotyping and RBC polymorphisms 21
10.8 Serology and Complement Studies 21
10.9 Meth Haemoglobin 22
10.10 Drug Concentrations 22
11 Sample Size 22
12 Endpoints 23

12.1 Efficacy endpoints 23
12.1.1 Primary Endpoint 23
12.1.2 Secondary Endpoints 23
12.2 Safety endpoints 23
13 Analysis 24
14 Trial Governance 24
14.1 Steering Committee (TSC) 24
14.2 Data Monitoring and Ethics Committee (DMEC) 25
15 Ethical considerations 25
15.1 Ethical Committee 25
15.2 Declaration of Helsinki 25
15.3 Informed consent 26
15.4 Withdrawing consent 26
16 Quality assurance 26
17 Publication Policy 26
18 Appendices 27
18.1 Warning signs of severe malaria 27
18.2 Definition of severe malaria 27
18.3 Definition of a serious adverse event 28
18.4 Treatment tables 29
18.5 SOP for acute hemolyses (modified from IMPROV study SOP version 2.0) 31
18.6 Malaria microscopy quality control procedures 36
18.7 Informed consent and assent 41

1 Executive Summary

Plasmodium vivax can form dormant liver stages that reactivate weeks or months following an acute infection. Recurrent infections can be associated with a febrile illness, a cumulative risk of severe anaemia, and even mortality. In co-endemic areas the risk of recurrence after both *P. vivax and P. falciparum* infections can be over 50% within 3 months. The only drug we have to kill *P. vivax* hypnozoites is primaquine which is currently given as a 14 day regimen. In Papua a retrospective study found very low effectiveness for unsupervised treatment. If true this has profound effects on treatment policy, suggesting that greater efforts are needed to encourage adherence to treatment.

We propose a cluster randomized, controlled, open label trial to assess the effectiveness of unsupervised versus supervised primaquine treatment in patients with uncomplicated malaria. Since the risk of recurrent *P. vivax* is high in patients with either *P. vivax or P. falciparum,* both infections will be included in the study. The study will be conducted in Mimika, in the southern part of Papua Province, Indonesia. Participants will be enrolled at village health posts and provided with schizontocidal treatment plus primaquine radical cure which will be either supervised or unsupervised depending on which cluster the clinic is in. Participants will be followed up for 6 months and assessed in regular intervals for the presence of patent and sub-patent malaria. The outcome of the study will contribute to an improved treatment scheme for uncomplicated malaria in this area.

Since any trial setting introduces a bias to treatment adherence the RCT will be complemented by a non-randomized observational cohort of patients with *P. vivax* infection who will be tested and treated according to national guidelines. Following treatment these patients will undergo the same follow up procedures as participants of the RCT.

2 Background and rationale

Malaria remains an important cause of illness in Papua, Indonesia. Our clinical and epidemiological studies in Papua (Indonesia) have shown the magnitude of malaria morbidity in infants in the first 5 years of life, including recurrent episodes of malaria, anaemia, malnutrition and coinfection[1,2]. Together these contribute significantly to

[1] Poespoprodjo et al, *Clin Infect Dis,* 2009
[2] Douglas et al, *BMC Med,* 2014

morbidity in early life, and almost certainly to the very high infant mortality rates in this region. Symptomatic illness and fatal disease is also seen throughout the whole of childhood and into adult life.

Recurrent episodes of malaria, both from *P. falciparum* and *P. vivax*, have significant haematological consequences. Deployment of a safe and effective radical cure is the key to vivax malaria elimination and has potential to reduce severe morbidity and even mortality in high transmission areas. This has been the focus on several clinical trials in this location, with a change of antimalarial policy to DHA-piperaquine (an Artemisinin Combination Therapy, ACT) in March 2006[3], and subsequent studies aimed at optimising dosing strategies. However blood stage treatments have no effect on the dormant liver stages (hypnozoites) that are formed by *P. vivax*. The only hypnozoitocidal drug that is available is primaquine (PQ), an 8-aminoquinoline (8-AQ), which can cause potentially severe drug-induced haemolysis in G6PD deficient patients[4]. The risk of haemolysis after primaquine depends on the dose administered and the erythrocyte G6PD activity of the individual exposed. The risk of serious haemolysis associated with G6PD deficiency makes clinicians reluctant to prescribe primaquine without prior testing, which is often unavailable[5]. When primaquine is prescribed, patient adherence to the standard 14 day regimen is poor, resulting in a high risk of relapse. A randomised comparative study showed that DHA-piperaquine (DP) plus supervised 14 days of primaquine in Indonesian soldiers returning from service in Papua with *P. vivax*, had 92% efficacy in reducing relapse over 12 months[6], highlighting that 14 days of supervised primaquine works well in this location. However in a recent observational study of 62,494 patients with vivax malaria attending the RSMM hospital in Papua, the efficacy of 14 days unsupervised primaquine compared to those who didn't receive any primaquine, was less than 15% (Manuscript under review). Although this was a non-randomised observational study, we hypothesize that this is due to poor adherence to the unsupervised regimen.

There are only two randomised clinical trials that have addressed the effectiveness of 14 day primaquine, comparing patients given supervised and unsupervised treatment[7,8]. Quantifying the adherence to treatment and its associated impact on the risk of recurrence

---

[3] Harijanto et al, *Acta Med Indones* 2010

[4] Von Seidlein et al, *Malar J,* 2013

[5] Ley et al, *Malar J,* 2015

[6] Sutanto et al, *Antimicrob. Agents Chemother*, 2013

[7] Leslie et al, *Trans R Soc Trop Med Hyg,* 2004

[8] Cheoymang et al, *Acta Trop* 2015

is a crucial step in focussing resources on developing novel approaches to improve effectiveness (such as education campaigns and directly observed therapy). Furthermore if recurrent malaria and anaemia contribute to mortality then interventions which reduce malaria may also reduce mortality.

High rates of *P. vivax* recurrence after *P. falciparum*

In Papua, recurrence rates of *P. vivax* are high after an initial infection, often reaching >70% within 3-4 months. *P. vivax* recurrence is also high after an initial mono-species infection with *P. falciparum.*

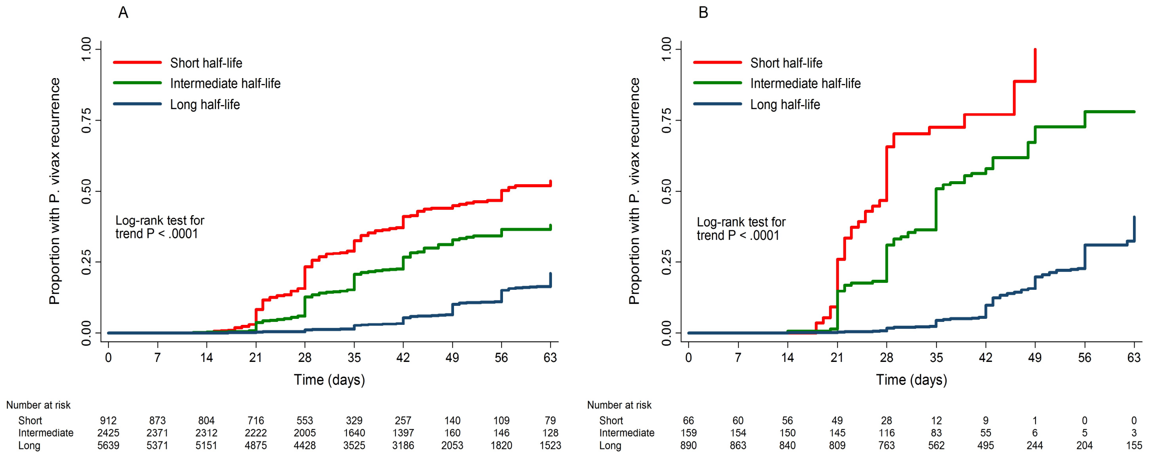

Figure 1. Risk of *P. vivax* recurrence after pure *P. falciparum* (A) and mixed species infection (B), following treatment with artemisinin combination therapy with fast and slowly elimination antimalarials (Douglas CID 2011).

After treating *P. falciparum* infections with highly effective ACTs, the risk of treatment failure is low for *P. falciparum* (>95% efficacy) but high for *P. vivax* recurrence. In fact the greatest risk of recurrence after falciparum malaria is *P. vivax* rather than *P. falciparum.* It is hypothesised that in co-endemic areas the fever of a *P. falciparum* infection stimulates the reactivation of the dormant *P. vivax* liver stages which are usual present but undetectable. Long half-life drugs provide prolonged post treatment prophylaxis to suppress the first relapse.

Similarly high rates of *P. vivax* recurrence after *P. falciparum* infection are also seen in Papua. This provides a strong rationale that in areas co-endemic for *P. vivax* and *P. falciparum*, radical cure with ACT plus primaquine would be highly effective and reduce the risk of recurrence of any malaria in the following 6 months.

3 Aim

We propose to assess the efficacy and effectiveness of radical cure in G6PD normal patients with uncomplicated malaria on reducing recurrent parasitaemia.

4 Study Objectives

Primary:

- To assess the effectiveness of a 14 day PQ treatment regimen in preventing recurrent *P. vivax* parasitaemia in G6PD normal patients with uncomplicated malaria

Secondary:

- To assess the efficacy of supervised or unsupervised PQ in preventing recurrent *P. vivax* (symptomatic and asymptomatic).
- To assess the efficacy of supervised or unsupervised PQ in preventing any recurrent parasitaemia
- To quantify the risk of *P. vivax* recurrence after *P. falciparum* infection in Papua.
- To quantify the comparative tolerability with supervised and unsupervised primaquine
- To identify determinants predictive of poor adherence to 14 days of PQ

5 Study design

5.1 Design and Rationale

This study is a health care facility based, cluster randomized, controlled, open label trial comparing supervised versus unsupervised primaquine treatment to prevent *P. vivax* parasitaemia in patients with acute malaria infection due to *P. falciparum* or *P. vivax*.

This trial aims to assess the effectiveness of unsupervised versus supervised primaquine treatment. In order to avoid spill over effects participants within one cluster will receive the same treatment.

The study is complemented by a non-randomized observational cohort to assess standard practice and determinants of treatment adherence.

5.2 Study site

The study will be carried out in Mimika district, in the lowlands of southern Papua, Indonesia. In this area multi-drug resistant falciparum and vivax malaria are equally prevalent. The region has adopted a universal policy of DHA-piperaquine, an artemisinin combination therapy (ACT), as the first line treatment for uncomplicated malaria due to all species. Malaria transmission is unstable with an annual incidence of 876 malaria episodes per 1000 person years. *P. falciparum* and *P. vivax* contributes approximately equally burden of disease[9].

Participants enrolment, follow up and treatment will be at village health posts (VHP). VHPs are the frontline maternal and child health care at village level. VHPs are a community based local initiative and supervised by community health centres at the sub-district.

5.3 Study population

Any participant above the age of 12 months fitting the inclusion criteria and living in one of the clusters of an established baby cohort study (the Baby Cohort study, UGM no: KE/FK/1023/EC, HREC no.: 13-2108) is eligible to participate.

5.4 Study duration and follow up period

The study will be conducted over a period of 24 months. With recruitment for 18 months and follow up periods for 6 months (table 1).

5.5 Cluster Randomization

Clusters (n=21) are based on an ongoing longitudinal study (the Baby Cohort study, UGM no: KE/FK/1023/EC, HREC no.: 13-2108). Individual clusters will be allocated randomly to one of two arms (unsupervised versus supervised radical cure) by an independent statistician.

[9] Karyana, *Malar J,* 2008

6 Treatment

6.1 Study treatment

6.1.1 Randomized arms

All participants will receive supervised dihydroartemisinin 2-4mg/ kg BW and piperaquine 16-32mg / kg BW (DHA-Pip) according to guidelines. The first three days of DHA-Pip will be supervised in all patients and if the participant vomits the treatment within 60 minutes a repeat dose will be administered.

The patient will be reviewed on day 2 after the third dose of DHA-Pip. If haemoglobin levels are above 9g/dL, the patient will be given primaquine (0.5mg/kg/day) for 14 days as per local guidelines for radical cure. According to the randomization cluster the primaquine regimen will be either unsupervised (current standard practice) or supervised on alternate days.

The same treatment allocation will apply for every symptomatic and asymptomatic malaria episode occurring during the follow up. During subsequent malaria episodes radical cure will be provided together with schizontocidal treatment. Schizontocidal treatment on day 2 and 3 will be unsupervised in the non-supervised arm during subsequent malaria episodes after enrolment.

The decision on whether or not to provide radical cure is based on the Hb value measured at the day of recurrence, a pregnancy test done at recurrence and the FST result obtained at enrolment. If a participant presents with an Hb level <9 g/dl, the participant will be treated with schizontocidal treatment only and asked to return every 14 days until the Hb is > 9g/dL and radical cure can be commenced.

Supervision of primaquine will occur at home (attended by a home visitor) or at the health post. Participants in the non-supervised treatment arm will receive standardized instructions on how to take treatment.

6.1.2 non-randomized cohort

Once the sample size within an unsupervised cluster has been reached, additional patients from the same cluster with a *P. vivax* or *P. falciparum* mono- or mixed infection will be enrolled into an observational cohort. All patients from this observational cohort will receive treatment according to national test and treatment guidelines (DHA-piperaquine plus 14

days primaquine (total dose 3.5mg/kg), no G6PD testing). The same treatment will apply for every symptomatic and asymptomatic malaria episode occurring during the follow up.
Patients from the observational cohort who are subsequently identified as being G6PD deficient will be censored from further treatment with primaquine for any subsequent *P. vivax* infection diagnosed during follow up.

6.2 Rescue treatment

Patients who fail to respond to the trial drug will be given according to national guidelines: a 7 day regimen of quinine plus doxycycline if older than 7 years and quinine plus clindamycin younger than 7 days old.
Patients who develop severe malaria will be admitted to hospital and treated with the recommended parenteral drug (intravenous Artesunate or quinine) according to national guidelines.

6.3 Concomitant treatment

At enrolment, all anti-malarial medication received in the preceding 4 weeks will be documented on the Clinical Record Form (CRF).
Regular medication at trial entry for conditions other than malaria, e.g. asthma, hypertension, etc. will be documented. Patients should continue to take these regular medications in the normal way.
Any additional drugs taken during the trial period for whatever reason must be documented (e.g. antibiotics for inter-current infection, anti-emetics, anti-pyretics etc.).
Patients who prematurely discontinue trial medication, or who fail to respond to trial medication and receive other anti-malarial therapy will have this documented with start and end date. Drugs with antimalarial activity should be avoided, unless prescribed by the attending clinician.

7 Socio-economic data collection

Understanding the reasons for poor treatment adherence and cost implications are critical information for policy makers to act on the results of the study. Quantitative and qualitative

data from participants, at community level and among health care providers involved in TRIPI on the perception of disease, treatment adherence and G6PD diagnostics and the associated cost implications will therefore be collected.

7.1 Quantitative Questionnaire

Quantitative data will be collected from all patients to gather socioeconomic data including those in the interventional and observational control arms on day 16 (at the end of primaquine treatment) or at a later time during the follow up. The questionnaire will be integrated into the case report form (CRF), and takes no more than 10 minutes. Collected data will include information on the socioeconomic status of the patient and their household, health seeking behaviour, factors of adherence and the household costs of illness incurred before and after treatment.

To estimate the cost per visit incurred at each health care centre when providing testing and treatment for malaria, 15 visits of patient interaction will be structurally observed for both the interventional and the observational arms. The patient's visit will be timed and the items used will be noted. If the patient is in contact with multiple health care workers, the time spent with each health care worker will be recorded individually. Laboratory procedures related to malaria diagnosis will be observed and items used will be noted. The time and cost spent due to the clinical trial will be excluded.

7.2. Qualitative interviews

In-depth interviews will be held with a purposive sample of approximately 100 respondents selected among (i) patients enrolled in the study, (ii) health care providers, and (iii) other community members. Interviews will take no more than 60 minutes and will only be conducted if prior informed oral consent has been provided (audiotaped). All respondents will be given an information sheet (attached) and provide verbal consent prior to interview.

Key areas that will be assessed among these respondents include: perceptions of malaria, perceptions and patterns of adherence, acceptance of the concept and practical implications of PQ based radical cure, additional structural and socio-economic factors affecting the effectiveness of treatment for *P. vivax* (including access to treatment, indirect and/or non-medical costs, alternative treatment seeking itineraries, delay in accessing the correct

treatment). Interviews among health staff in addition will include topics on acceptance of the concept of primaquine based radical cure for all malaria species and practical implications of PQ based radical cure for *P. falciparum,* acceptability and adherence to G6PD point of care test results, and translation of G6PD tests into treatment decisions.
Interview guides will be continuously adapted based on the preliminary results emerging from the thematic analysis of transcript data.

8 Study procedures

8.1 Study procedures for the randomized arms

The study procedures are summarised in Table 1

8.1.1 Enrolment

Patients attending one of the studies village health posts (VHP) will be screened for inclusion criteria. All eligible patients will be informed about the study and asked to participate. Women between the age of 14 to 49 willing to participate will be asked to take a pregnancy test. If patients are eligible and willing to participate they will be asked to sign a written informed consent form for enrolment. In the case of minors the legal guardian will be asked to sign on behalf of the minor. Minors above the age of 13 will be asked to sign an assent form.

8.1.1.2 Inclusion Criteria

- Infection with *Plasmodium falciparum* or *P. vivax* either alone or mixed
- Age >12 months
- Weight >5kg
- Living in the study clusters (Baby Cohort study, UGM no: KE/FK/1023/EC, HREC no.: 13-2108)

8.1.1.3 Exclusion Criteria

- General danger signs or symptoms of severe malaria[10]
- Anaemia, defined as Hb <9g/dl
- G6PD deficiency (as determined by FST)
- Pregnant women as determined by Urine β-HCG pregnancy test

[10] WHO, 2015

- Known hypersensitivity to any of the drugs given

8.1.2 Day 0

At enrolment a brief questionnaire will be completed including demographic information and medical history and a physical examination will be conducted. Venous blood will be collected in all consenting participants above the age of seven, and capillary blood in all others. Collected blood will be used for a malaria slide, a fluorescent blood spot test (FST) to determine G6PD activity and Hb measurement. An aliquot of blood will be stored in a microtainer™ for later molecular examinations for parasite and host PCR/genotyping and serology (see Laboratory procedures, section 8). All participants will then receive the first dose of supervised schizontocidal treatment.

8.1.3 Day 1

All participants will be reviewed, for symptoms and a brief physical exam. Capillary blood for a malaria slide will be collected and the second dose of schizontocidal treatment administered.

8.1.4 Day 2

All participants will be reviewed, for symptoms, adverse events and a brief physical exam. Capillary blood for a malaria slide will be collected and the third dose of schizontocidal treatment administered. If the Hb of the participant is above 9 g/ dL and the FST test from D0 indicates a G6PD normal result the participant is eligible for radical cure. Participants with a G6PD deficient result will be informed of their status, and will be provided with information on G6PD deficiency and its consequences in everyday life; they will be excluded from further activity in the study. The course of primaquine will be either unsupervised (current practice) or semi-supervised – according to the cluster of residence. Those receiving unsupervised treatment will be provided with the full treatment course for radical cure and instruction on how to take the prescribed treatment. Participants are asked to return to the VHP if they feel unwell. Participants of the supervised arm will take their first PQ dose.

8.1.5 Days 3-15 (only participants in the supervised arm)

Participants in the supervised arm will be reviewed on alternate days for directly observed treatment with primaquine. If any evidence of haemoglobinuria (dark urine) or adverse events, a capillary sample will be taken for Hb measurement.

Patients in the unsupervised arm will not be reviewed unless they present with complaints.

8.1.6 Day 16

On the day after the last dose of the primaquine treatment, participants in both groups will be reviewed with a symptom questionnaire and physical exam. Capillary blood will be collected for Hb measurement, a blood film and storage of up to 300ul in a microtainer for parasite PCR, and drug concentration measurement. Meth-Haemoglobin will be measured using a non-invasive finger probe (Masimo™, USA). A pill count will also be conducted in participants allocated to the unsupervised primaquine arm.

8.1.7 Months 1-6

Participants will then be reviewed again on day 28 and monthly thereafter, with a symptom questionnaire and brief physical exam. A capillary blood sample will be taken for Hb measurement (which will be reported back to the clinician and patient), a blood film and 200ul blood stored in a microtainer. On months 1, 3 and 6 a sample will be stored for later serological analysis.

8.1.8 Day of recurrence

All participants with fever or symptoms indicative of malaria will be asked to return to the same VHP as on enrolment. A symptom questionnaire will be filled out and brief physical examination will be done. A capillary blood sample will be collected and a blood film will be taken for malaria diagnosis (which will be read immediately), haemoglobin measurement, repeat G6PD testing, and the remaining blood will be stored in a microtainer (for parasite PCR and serology).Those older than 7 years with microscopy confirmed malaria will be asked to provide a venous sample. Female participants will be asked to undergo a repeat pregnancy test and if pregnant will be excluded from further primaquine treatment and referred to an antenatal clinic for further follow up. If Hb levels are above 9g / dL and the participant is not pregnant he/she will receive schizontocidal treatment and primaquine radical cure; both treatments will be started immediately. Treatment will be either

unsupervised or supervised according to the initial randomization cluster. The last visit for the episode will be on day 14 after start of radical cure. The participant will then be reviewed accordingly to their monthly schedule determined at enrolment.

If the Hb at recurrence <9g / dL the participant will only be treated with schizontocidal treatment and will be asked to return on a biweekly (every 14 days) basis until the Hb level is >9g / dL and radical cure can be commenced.

8.2 Procedures for serious adverse events

All participants with SAEs[11] (defined in Appendix section 16.4) will be reviewed with a standard questionnaire. Patients suspected of severe malaria or sepsis will be treated according to local hospital guidelines.

All patients with an SAE will be assessed to ascertain malaria and haematological status. Venous or capillary blood will be taken for Full Blood Count (FBC), and if clinically indicated Urea and Electrolytes (UEC and Liver Function Tests (LFTs)). Relevant investigations will be ordered according to the attending physician. All results of clinical investigations will be collated on an SAE report.

8.3 Discontinuation/ Withdrawal of Participants from Study Treatment

Each participant has the right to withdraw from the study at any time. The investigator will withdraw a participant and stop further administration of study drugs if the participant requires a blood transfusion or has a fall in Hb below 7g/dL.

Warning signs: In addition, a participant may, at the discretion of the attending physician, be withdrawn from the study at any time if the investigator considers it necessary for any reason including:

- Macroscopic haemoglobinuria (i.e. from red to black urine) detected by a visual inspection of urine collected in a white cup (during radical cure), associated with a fall in Hb by >25% (during radical cure)

[11] SAEs are defined as unwanted medical events resulting in death or hospitalization, being life-threatening or resulting in disability or permanent damage, congenital anomalies or requiring intervention to prevent permanent damage

Other reasons for exclusion/withdrawal:

- An adverse event which requires discontinuation of the study medication or results in inability to continue to comply with study procedures
- Consent withdrawn
- Lost to follow up for 2 or more monthly visits

If the participant is withdrawn due to an adverse event, the investigator will arrange for follow-up visits or telephone calls until the adverse event has resolved or stabilised.

9 Study procedures for the non-randomized observational cohort

All participants will have to provide written informed consent prior to enrolment. In order to be eligible the following enrolment criteria apply:

9.1 Inclusion Criteria

- Infection with *P. vivax* either alone or mixed
- Age >12 months
- Weight >5kg
- Living in the study clusters (Baby Cohort study, UGM no: KE/FK/1023/EC, HREC no.: 13-2108)

9.2 Exclusion Criteria

- General danger signs or symptoms of severe malaria[12]
- Anaemia, defined as Hb <9g/dl
- Pregnant women as determined by Urine β-HCG pregnancy test
- Known hypersensitivity to any of the drugs given

Upon enrolment the same procedures as for routine care patients are undertaken. At the end of radical cure (day 14) all patients will undergo the same procedures as patients from the RCT to ensure safety of the participant. All subsequent follow up procedures are similar to procedures from the RCT.

[12] WHO, 2015

Patients tested G6PD deficient in the course of the follow up will be removed from the cohort in case of a relapse (table 1).

Table 1: Treatment and sampling strategy

Time point after Enrolment	D0	D1	D2	D3-15	D16	D28***	M2***	M3***	M4***	M5***	M6***	Day of recurrence
**Treatment**												
**Schizontocidal treatment**	X	X	X									X**
**Radical cure#**				X**	X**							X**
**Procedures**												
**Symptom Questionnaire**	X	X	X	X**	X	X	X	X	X	X	X	X
**Medical Examination**	X	X	X		X	X	X	X	X	X	X	X
**Pregnancy test**	X											X
**Capillary blood collection**	X	X	X	X**$	X	X	X	X	X	X	X	X
**Venous blood collection***	X											X
**G6PD diagnosis**	X&	(X)%	(X)%	(X)**$%	(X)%	(X)%	(X)%	(X)%	(X)%	(X)%	X	(X)%
**Blood film**	X	X	X		X	X	X	X	X	X	X	X
**Hb**	X		X		X	X	X	X	X	X	X	X
**Parasite PCR/Genotyping**	X				X	X	X	X	X	X	X	X
**Host genotyping & RBC polymorphism**	X											
**Serology**	X					X		X			X	X
**Pill Count**					X							
**Met Hb**					X							
**Drug Concentrations: Primaquine and Carboxyprimaquine**					X							

*only in consenting children above the age of 5 years; **Supervised arm only; ***Home visit or clinic review
# For recurring episodes radical cure will be on days 0-14
$ only if adverse event with fever or haemoglobinuria
& not in observational cohort
% repeat testing of G6PD will be undertaken providing sufficient blood sample remaining after other tests

10 Laboratory procedures

10.1 Urine β-HCG pregnancy test

A urine β-HCG pregnancy test will be conducted on all women aged 13-49. Anyone testing positive will be excluded. Pregnancy testing will be performed at screening and before a recurrent *P. vivax* episode is treated with primaquine. In case the patient is pregnant PQ will be withheld.

10.2 Blood collection

Up to 300µl of capillary blood will be collected via a finger / heel prick on day 0 and 2, at the end of radical cure (day 16), and then monthly until the end of follow up and on every unscheduled visit. Capillary blood will be stored in EDTA microtainers at 4-8°C until further processing.
Up to 8ml of venous blood will be collected in EDTA vacutainers from all participants above the age of 5 years at day of enrolment and recurrence. Whenever venous blood is collected no capillary blood will be collected.

10.3 G6PD deficiency testing

A fluorescent spot test (FST) will be performed prior to enrolment at a reference centre in Timika. Negative (no fluorescence) and intermediate test results will be categorised as G6PD deficient. Patients with a G6PD deficient FST results are excluded from the randomised study but included in the observational cohort of standard practice. Positive and negative controls will be run at regular intervals to ensure the quality of the test result. The result will be recorded and the test will not be repeated for subsequent malaria episodes. G6PD status will also be assessed using quantitative and qualitative assays whenever capillary blood is collected and sufficient blood from the 300µl is available.

10.4 Malaria Microscopy

Slides for microscopy will be collected at enrolment, day 1, 2, at the end of radical cure, on months 1-6 and on any unscheduled visits. Quality control and assurance will be based on procedures as outlined in the appendix (section 15.4).

10.5 Haemoglobin

Haemoglobin (Hb) will be measured at baseline, day 16, monthly, if haemoglobinuria is reported and on any unscheduled visit. Hb will be measured using a Hemocue™ Hb (Angelholm, Sweden) machine or a Carestart Hb machine (Carestart, USA).

10.6 Parasite molecular analysis

Whenever blood is collected a sample will be stored in a microtainer for parasite detection by PCR and genotyping. Sub-patent infections (PCR positive but blood film negative) are common and can results in anaemia and increased risk of recurrence. Parasite DNA will be extracted from these samples for detection of sub-patent parasitemia. All Plasmodium *spp.* detected will be genotyped for finger printing (to determine new or different parasites from previous or latter infections), genetic diversity and known and putative molecular markers of drug resistance, including but not limited to: *pfmdr1*, p*fcrt, Kelch, pvmdr1 and pvcrt.* Molecular analysis of the parasite will be done at the Eijkman Institute for Molecular Biology in Jakarta, Indonesia, or by sequenom analysis at the Sanger Institute in the UK.

10.7 Host genotyping and RBC polymorphisms

Human DNA will be extracted from dried blood spots collected at baseline. All samples will be assessed using molecular techniques for known and unknown variants of the G6PD gene (Xq28) and other candidate markers related to malaria susceptibility and treatment outcome including alpha and beta thalasaemia, Gerbich and Ovalocytosis.

In collaboration with the Eijkman Institute in Jakarta, Indonesia, red blood cell polymorphisms will be assessed by molecular analysis.

CYP2d6 polymorphisms which are known to affect primaquine metabolism will be documented. Where possible all samples will be processed within Indonesia, however additional sequencing may require transfer to the Sanger Institute, UK, under appropriate material transfer agreement and according to national guidelines.

10.8 Serology and Complement Studies

Serum collected at enrolment will be assessed for antibodies against a standard panel of recombinant antigens for both *P. falciparum* and *P. vivax*. The panel will be chosen to represent all stages of the parasite life cycle and encompass both genetically conserved and variant antigens. Antibody levels will be determined by high-throughput enzyme-linked immunosorbent assay (ELISA) and flow cytometry together with functional growth inhibition

and phagocytosis assays to assess functional immunity. Micro array analysis will be undertaken in a subset. In addition analysis of complement function will be assessed by ELISA. These assays can be achieved using minute quantities of sera from finger prick samples. The respective procedures will be performed at Eijkman Institute in Jakarta, The University of Gadja Mada, Yogjakarta, or the Burnet Institute, Melbourne, Australia.

10.9 Meth Haemoglobin

Methhaemoglobin is induced by derivatives of primaquine and hence an indicator of drug exposure. Methhaemoglobin will be measured using a Masimo Rad – 57 on a non-invasive basis on site.

10.10 Drug Concentrations

Blood to measure drug concentrations will be collected on day 16, at the end of radical cure. The relationship between final primaquine and carboxyprimaquine concentrations and the risk of subsequent *P. vivax* recurrence will be explored and correlated with Meth Hb results. Drug concentrations will be measured at the Mahidol Oxford Research Unit, Bangkok, Thailand.

11 Sample Size

A total of 21 clusters with 420 participants (20 participants from each cluster) will be needed to detect an absolute reduction of 20% in the incidence risk of *P. vivax* malaria recurrence from 30% in the unsupervised primaquine arm to 10% in supervised primaquine arm, detected during the 6 months following their first episode of *P. vivax* or *P. falciparum* infection. This sample size will have 90% power at the two-sided 5% significance level (assuming 15% loss to follow up, and a conservative intra-cluster correlation coefficient (ICC) of 0.05). The latter was chosen as a conservative estimate, following documented ICC of 0.018 in a cluster RCT of IPT in children in Senegal.

In addition 220 participants with a *P. vivax* mono- or mixed infection will be enrolled into an observational cohort to address secondary objectives on treatment adherence.

12 Endpoints

12.1 Efficacy endpoints

12.1.1 Primary Endpoint

- The incidence risk of the first recurrent episode of microscopic *P. vivax* malaria over 6 months in patients enrolled with any malaria infection.

12.1.2 Secondary Endpoints

- The incidence risk of the first recurrent episode of *P. vivax* malaria over 6 months in patients enrolled with *P. vivax* malaria.
- The incidence risk of the first recurrent episode of *P. vivax* malaria over 6 months in patients enrolled with *P. falciparum* malaria.
- The incidence rate of all episodes of *P. vivax* malaria over 6 months in patients enrolled with malaria due to *P. falciparum* or *P. vivax*.
- The incidence rate of all episodes of *P. vivax* malaria over 6 months in patients enrolled with *P. vivax* malaria.
- The incidence rate of all episodes of *P. vivax* malaria over 6 months in patients enrolled with *P. falciparum* malaria.

12.2 Safety endpoints

- The proportion of patients vomiting their medication within 1 hour of administration.
- The proportion of patients vomiting any of their primaquine doses during the 14 day supervised course.
- The proportion of adverse events and serious adverse events over 6 months in all patients.
- The incidence risk of severe anaemia (Hb<7g/dl) and/or the risk for blood transfusion over 6 months.
- The incidence risk of an acute drop in Hb >5g/dl compared to baseline or fractional fall >25% in Hb to less than 7 g/dl within 14 days of starting primaquine.

13 Analysis

The efficacy analysis will be based on a *modified intention to treat* population. A conservative analysis will be repeated on the *per protocol* population which will exclude patients with major protocol violations. The safety data will be analysed using safety data population which comprised all patients that received at least one dose of treatment.

The incidence risk (cumulative risk) will be estimated by survival analysis with the (Kaplan Meier method). Comparisons between treatment arms will be presented as Hazard Ratios using a Cox regression model.

The total number of symptomatic and asymptomatic *P. vivax* episodes occurring during the 6 months follow up will be presented as incidence estimates per person year of observation (PYO) per treatment arm. Incidence rates will be calculated by dividing the number of *P. vivax* episodes by the number of person-years of observation (PYO) in the study population. On the individual patient level the start date for PYO is the day of enrolment into the study, stop date is the last visit performed (either completed study at 6 months or any last visit before lost to follow up and/or censoring). The period between start and stop dates for each patient will be calculated in days and divided by 365 to determine PYO, which will then be summed for all participants. Comparison between incidence rates will be presented as Incidence Rate Ratios (IRR) calculated using a negative binomial regression model.

Safety data will be presented as summary statistics (including number, mean, median, interquartile range, standard deviation, minimum and maximum) of all vital signs variables. Values and changes from baseline especially for haemoglobin will be calculated for each visit by treatment group.

Qualitative data analysis will be a retroductive process, combining an emergent theory process with concurrent data collection.

14 Trial Governance

14.1 Steering Committee (TSC)

The TSC will monitor the trial implementation in terms of protocol adherence, trial integrity and ensuring patient safety. The team is proposed to be chaired by independent individual from The Indonesian Ministry of Health: Chair: Dr. Elizabeth Jane Soepardi, MOH, Director of

Child Health. The other members: 1) Dr. Asik Surya, MPPM, Independent member, Head of National Malaria Control Program, MOH; 2) Dr Rini Poespoprodjo (PI); 2) Prof. Yati Soenarto, Supervisor, Gadjah Mada University, Indonesia; 3) Dr. Ida Savitri Laksono, Head of Infesctious Diseases, Gadjah Mada University, Indonesia; 4). Dr Bill Hawley, Chairperson, Malaria Programme manager, UNICEF, 5) Dr Ferdinand Laihad/Martin Weber, independent member, UNICEF/WHO; 6) Prof Ric Price, co-investigator. The trial statistician, site PIs and trial managers will be co-opted. TSC meetings will be held annually.

14.2 Data Monitoring and Ethics Committee (DMEC)

The trial will have an independent DMEC consisting of 3 independent members: drug safety experts, paediatrician, independent statistician and clinical trial expert. The potential members will be discussed at the trial coordination meeting. They will meet by teleconference 6 monthly to review the data from an ethical standpoint, to ensure the safety, rights and well being of trial participants.

15 Ethical considerations

15.1 Ethical Committee

This study will be submitted for formal review and approval to the Institutional Review Board of the Menzies School of Health Research, the Medical Health Research Ethics Committee Faculty of Medicine Universitas Gadjah Mada, Indonesia and Eijkman Institute of Molecular Biology, Indonesia. No participant will be enrolled or samples processed before written approval from these bodies is obtained.

15.2 Declaration of Helsinki

The study will be carried out according to the principles stated in the Declaration of Helsinki (Ethical Principles for Medical Research Involving Human Subjects) as amended in 2008, all applicable regulations and according to established international scientific standards.

Any substantial amendments to the protocol or the Informed Consent Form will also be submitted for approval to the same ECs and competent authorities and will be implemented only after approval has been obtained.

15.3 Informed consent

The information and consent form will be translated to Bahasa and back translated to English to ensure adequate translation. Written informed consent will be obtained from all participants or their legal guardians. Information provided during the consenting process will include description of the sample collection procedure, aim of the study, details on the data collected, potential benefits and risks and assurance of confidentiality for all information and results generated by the study. Legal representatives of enrolled children will be asked for written informed consent.

Information and consent form will be read out to all participants not fully literate; illiterate participants willing to participate will provide consent by a fingerprint in the presence of a witness.

15.4 Withdrawing consent

All participants will be thoroughly informed about their right to withdraw consent at any time without having to provide a reason for withdrawal or having to fear negative consequences.

16 Quality assurance

Quality assurance and monitoring will be conducted in regular intervals. Procedural and reagents controls will be provided throughout the study period. Performance of laboratory technicians is assured by on-site training and site visits. Site-visits are foreseen by the investigators on a regular basis, as well as weekly e-mail or Skype communication with local study staff.

17 Publication Policy

All Investigators will be involved in reviewing drafts of the manuscripts, abstracts, press releases and any other publications arising from the study. Authorship will be determined in accordance with the ICMJE guidelines and other contributors will be acknowledged.

18 Appendices

18.1 Warning signs of severe malaria

- Not able To Drink/Eat
- Vomiting Excessively
- Recent History Of Convulsions
- Altered Mental State
- Unable To Sit/Stand Up

18.2 Definition of severe malaria

One or more of the following clinical or laboratory features classifies the patient as suffering from severe malaria (adapted from the 2010 WHO guidelines) and should be excluded from the study:

- impaired consciousness or unrousable coma
- prostration, i.e. generalized weakness so that the patient is unable walk or sit up without assistance
- failure to feed
- convulsions
- deep breathing, respiratory distress (acidotic breathing)
- circulatory collapse or shock, systolic blood pressure < 80 mmHg in adults and <70 mmHg in children
- clinical jaundice
- observed / history of haemoglobinuria
- abnormal spontaneous bleeding
- reliable history of anuria or oliguria over the last 24 hours

If measured or done:

- hypoglycaemia <2.2 mmol/L (or <40 mg/dl)
- metabolic acidosis (plasma bicarbonate < 15 mmol/l)
- hyperlactataemia (plasma lactate > 4 mmol/L)
- renal impairment (serum creatinine > 265 µmol/l)
- pulmonary oedema on X-ray

18.3 Definition of a serious adverse event

A serious adverse event (SAE) in human drug trials is defined as any untoward medical occurrence that at any dose

- results in death,
- is life-threatening
- requires inpatient hospitalization or causes prolongation of existing hospitalization
- results in persistent or significant disability/incapacity,
- is a congenital anomaly/birth defect, or
- requires intervention to prevent permanent impairment or damage

18.4 Treatment tables

Schizontocidal Treatment

Tablets containing 40 mg dihydroartemisinin and 320 mg piperaquine

Body weight (kg)	Number of tablets		
	Day 0	Day 1	Day 2
=5	1/4	1/4	1/4
6 - 10	1/2	1/2	1/2
11 - 17	1	1	1
18 - 30	1 ½	1 ½	1 ½
31 - 40	2	2	2
41 - 60	3	3	3
61 - 80	4	4	4
81 - 100	5	5	5

Rescue medication. Patients who fail to respond to the trial drug will be given unsupervised quinine and clindamycin treatment for 7 days (Dose: quinine 10 mg/kg body weight 3 times/day and clindamycin 5 mg/kg body weight 3 times/day). Parasitological and clinical response will be checked at day 3 and 7 after the commencement of oral quinine therapy.

If severe complication(s) occur, intravenous artesunate (2.4 mg/kg bodyweight at 0, 12 and 24 hours) or quinine drips (10 mg/kg bodyweight 8 hourly) must immediately be given along with the required supportive treatments according to the hospital protocol.

Hypnozoitocidal treatment

Primaquine (PQ) (each tablet contains 15 mg primaquine) will be given daily for 14 days with food (daily target dose 0.5mg / kg BW):

Weight (kg)	Total daily dose (mg / kg BW)	Number of tablets (15mg) per day over 14 days	Total does received (mg / kg BW)
5.0-24.9	0.3 - 1.5	0.5	4.2 - 21
25.0-34.9	0.4 - 0.6	1	5.6 – 8.4
35.0-45.9	0.5 – 0.6	1.5	7.0-8.4
≥46.0	≤0.6	2	≤8.4

18.5 SOP for acute hemolyses (modified from IMPROV study SOP version 2.0)

Introduction

This Standard Operating Procedure (SOP) outlines the approach to managing suspected haemolysis in trial participants. The key points are repeated in a one-page flowchart for clarification. There are two annexes that contain additional information on the symptoms, signs and investigation of anaemia and drugs that can cause haemolysis or interact with primaquine.
The clinical judgement of the attending clinician and patient safety should always come first, regardless of the trial protocol. Each site varies in capacity, hence management and interventions should be adapted to local clinical practice.
Any concerns should be raised by the principal investigators at each site with the trial co-ordination team.

Background

Haemolysis in acute malaria is inevitable, the mean haemoglobin concentration usually falling within the first week, with recovery usually taking 28-42 days. The use of primaquine in patients with *P. vivax* infection can cause additional haemolysis particularly in patients with glucose-6-phosphate dehydrogenase (G6PD) deficiency; the predominant mechanism for this is intravascular haemolysis. Clinicians need to be vigilant for the signs and symptoms of haemolysis and the risk of trial subjects becoming anaemic. Severe haemolysis and anaemia in a patient in this study is likely to result from a variety of factors, including: the severity of G6PD enzymatic deficiency, the dose of primaquine, the degree of parasitaemia and nutritional status.

Identification of acute haemolysis

At each clinical encounter the clinician should exclude the possibility of significant haemolysis. This
includes:
1. ***Detailed history and examination***: including a history of dark urine, jaundice, breathlessness and
other symptoms of anaemia. A full drug history should be taken looking in particular for the use of drugs and herbal remedies that can cause haemolysis or interact with primaquine
2. HemoCue haemoglobin measurement: this should be compared against previous haemoglobin
results, particularly the baseline value prior to starting primaquine, to gauge the degree and speed
of decline.
3. ***Hillmen urine colour estimation for haemoglobinuria***: urine should be placed in a clear glass container and held up against a white piece of paper, in a well illuminated area, before estimating

the colour compared to the Hillmen Colour Chart. Urine colour estimation should be carried out as
soon after voiding as possible. A score of 5 or above is considered evidence of haemoglobinuria*.*

4. Repeat G6PD fluorescent spot test: if on the basis of clinical assessment, haemoglobin measurement and urine colour assessment for haemoglobinuria, it is considered that haemolysis
may be occurring. All patients in the G6PD normal arm of the study should have a repeat G6PD fluorescent spot test to confirm correct allocation to the G6PD normal arm.

5. *Intensive review*: any concern should prompt increased frequency of review or admission to
hospital for monitoring at the discretion of the attending clinician.

Based on this assessment, patients should be categorised into:
i) those in whom there is currently no major concern for haemolysis.
ii) those who have indicators of clinical concern but who do not constitute severe haemolysis
iii) those with severe haemolysis or anaemia

The decision to retreat subsequent *P. vivax* infections with primaquine in patients who have experienced an episode of acute haemolysis, is at the discretion of the attending clinician and is **not** a protocol requirement. Patients who have had an episode of severe acute haemolysis related to primaquine use, are at significant risk of further haemolysis on reexposure to primaquine or other medications causing oxidative stress. Hence the decision to redose should be made after assessing the risks and benefits and discussing these with the patient/guardian.

The following are suggested guidelines :
If the haemoglobin falls by ≥ 1 g/dL in the preceding 24 hours: primaquine should continue to be withheld and the patient continued to be reviewed closely.
If the haemoglobin is stable or falls by < 1 g/dL in the preceding 24 hours: the patient can be re-dosed with primaquine, at the discretion of the attending clinician. Further daily monitoring should be undertaken for at least 3 days. Patients should continue as per protocol to complete follow up.
If the patient has clinical decompensation associated with recent primaquine use and/or anaemia:
then the patient should be admitted to hospital for blood transfusion. The current primaquine
regimen should be stopped.

Repeat G6PD testing
Patients with a haemolytic SAE or warning signs should have their G6PD status rechecked immediately. Samples should be taken prior to blood transfusion (since transfusion can mask G6PD deficiency) as well as 6 weeks after the resolution of the severe acute haemolysis (or 12 weeks after the receipt of a blood transfusion).

Subsequent Clinical Management
Patients without clinical compensation who restarted their primaquine treatment without further

deterioration should complete their primaquine and continue in the study as per the study schedule.
If the patient's primaquine regimen is stopped they should be encouraged to remain in the study and continue to be followed up. If patients develop subsequent symptomatic recurrent *P. vivax* parasitaemia, they should be considered for primaquine regimen on a case by case basis.

MANAGEMENT OF ACUTE HAEMOLYSIS

Any clinical concern of acute haemolysis (e.g. symptoms of anaemia, dark urine, clinical jaundice or falling haemoglobin) should be trigger the following:

1. A thorough history and examination
2. Immediate HemoCue haemoglobin measurement
3. Hillmen urine colour estimation for haemoglobinuria
4. Repeat G6PD test if randomised to the G6PD normal arm to confirm correct randomisation
5. Contact Bangkok coordinating team

INDICATIONS TO STOP PRIMAQUINE /PLACEBO

Either of the following:

1. Referred for blood transfusion
2. Anaemia (Hb<7g/dl) with clinical decompensation

Continue to assess daily or more frequently and manage as indicated or until resolved.

Refer to Bangkok coordinating team to discuss the need to unblind

Continue routine follow up for **FULL** year from randomisation.

INDICATIONS TO WITHOLD PRIMAQUINE/ PLACEBO:

Any one of the following:

1. Hb <7 g/dL & daily activities unaffected
2. Macroscopic haemoglobinuria (Hillmen ≥ 5)
3. Fractional haemoglobin fall ≥ 25% from baseline
4. Other significant clinical concern of anaemia or haemolysis

Daily clinical review and Hb measurement

If Hb falls ≥ 1 g/dL over preceding 24 hours:

Withhold primaquine/placebo

Continue review and repeat Hb daily until the patient can either recommence primaquine/placebo or needs referral for blood transfusion

If Hb stable or drop over preceding 24 hours is < 1 g/dL:

Re-dose with primaquine/placebo (at the discretion of the attending clinician)

Review and repeat Hb daily until Hb stable for 3 days.

SAEs NOTIFICATION WITHIN 24 HOURS FOR:

1. Hb < 7 g/dL
2. Referral for blood transfusion
3. Macroscopic haemoglobinuria (Hillmen ≥ 5) + fractional haemoglobin fall ≥ 25% from baseline

AE FORM TO BE COMPLETED

1. All patients who have primaquine withheld for any reason

Further guidelines for the management of patients with a haemolytic SAE

Patients with a haemolytic SAE and clinical decompensation should be admitted to a hospital with a blood transfusion service and managed according to local protocols.

The key principles of managing acute drug-induced haemolysis include the following:

1. Stopping all drugs known to cause haemolysis
2. Comprehensive clinical evaluation and intensive monitoring, including vital signs, fluid balance and frequent assessment for haemoglobinuria
3. Monitoring of haemoglobin and renal function
4. Blood transfusion if indicated

The following guidelines are offered as suggested indications for transfusion:

Haemoglobin < 7 g/dL and clinical decompensation due to anaemia:

- breathless at rest
- breathlessness on exertion over a short distance
- severe fatigue/lethargy/dizziness
- prostration
- signs of cardiac failure
- cardiac chest pain

18.6 Malaria microscopy quality control procedures

**Title:**	**Quality control for malaria microscopy**	
Version number	1.0	
Effective date	11 October, 2021	
Author	Benedikt Ley	
Approved by	Jutta Marfurt	
Document history		
Version	Effective date	Changes
1.0	10 August 2015	NA

1. PURPOSE AND BACKGROUND

Aim of this SOP is to provide standardized procedures that assure high quality malaria microscopy data.

Correct and high quality malaria microscopy data is of vital importance for the health and safety of patients providing blood for malaria diagnosis and for all research related activities.

There are a number of factors that affect the reading of malaria slides and hence, good quality data. These range from supplies used, methods of slide preparation to degree of training of the microscopist.

This SOP does not provide a tool to assess these individual components, but to monitor and control the outcome of malaria microscopy.

2. RESPONSIBILITY

Ensuring that slides are prepared with suitable consumables using appropriate techniques and are interpreted by well-trained microscopists is the direct responsibility of the head of laboratory on site.

3. PROCEDURES

A) <u>Internal</u> Quality Control (IQC)

A.1) Requirements that must be met:

- All slides must be prepared and read according to standardized local SOPs.
- A specific person responsible for quality control of malaria microscopy must be identified and appointed (e.g. head of laboratory).

A.2) When does double reading apply?

- Double reading must be performed for the first six months of a new project or if a new first or second reader starts on the project / is employed.

- If after any six months the following is observed after EQC review:
    - A parasite density discrepancy rate of ≤50%
    - A species discrepancy rate of ≤10%
    - A positive / negative discrepancy rate of ≤10%

AND

- All readers are certified according to WHO criteria at level ≥2[13].

…double (and triple) reading can be reduced to 20% of all slides.

A.3) Procedure for double reading:

- All slides must be read by at least two independent and well-trained microscopists.
    - Microscopists must be blinded towards the other reading result.

- Results must be entered into the "Obare method calculator" to be assessed for consistency.
    - If the calculator does not propose a third reading, the calculated mean value is recorded as the final result.
    - In case of significant discrepancies, the calculator will suggest a third reading.

- The third microscopist must be blinded towards the first two results.

---

[13] WHO & TDR: *Microscopy for the detection, identification and quantification of malaria parasites on stained thick and thin blood films in research settings*, 2015 [online]: http://www.wwarn.org/sites/default/files/attachments/procedures/microscopy_eng.pdf] (last accessed 17.06.2015)

- If the third reading is within a reasonable range of the first or second reading, the calculator will calculate the mean parasite density of the two most congruent values and this mean value is recorded as the final result.

- If all three readings are discordant (see Appendix I), the entire process needs to be repeated. If all three readings remain discordant, all three results must be recorded as preliminary results and the supervisor must be informed immediately. The supervisor will provide additional assistance and initiate further actions as required.

B) External Quality control (EQC)

B.1) Requirements that must be met for this procedure

- The EQC reader must be a microscopist at expert level according to WHO criteria.
- The EQC reader must follow the same SOP for slide reading as previous readers at the site.
- The EQC reader must be blinded to all previous results.
- The EQC reader must be independent of the institution and context in which the slides were collected.

B.2) Procedures

- At least the following slides must be shipped to the institution appointed as EQC center:
    - Every slide collected during the first visit of a patient.
    - Every slide collected at the first visit of a recurrent malarial episode.
    - 10% randomly selected slides read by readers 1, 2 and 3.
    - All slides which needed a 3rd reading.

- Selected slides must be sent to the EQC center with no other information apart from:
    - Study code (Timika studies : “STUDY”)
    - Study identification number (Timika studies : “CODE”)
    - Date of sample collection (Timika studies : “DATE”)

- Slides are read and results reported back to the appointed local person in charge of malaria microscopy QC.

- The EQC center will also report back on slide quality (i.e., thick film size, thickness, staining, artifacts: yes/no; thin film monolayer: yes/no, staining artifacts: yes/no)

- All readings provided by the EQC center are entered into the “Obare method calculator” and compared to the estimated mean calculated for IQC purposes. All results are recorded as the:
    - fraction of all slides assessed with discordant negative / positive results
    - fraction of all slides assessed with discordant parasite densities
    - fraction of all slides assessed with discordant speciation results
    - fraction of all slides assessed with discordant mono-species infection / mixed species infection results

- In addition, summary statistics on the slide quality must be reported, as well as the proportion of slides for which no remarks with respect to film quality, staining, etc. have been made

- The results of the EQC center must be discussed with the microscopy team as soon as possible and depending on the outcome, appropriate actions must be taken.

Procedure to define discordant results if the "Obare methods calculator" is not available:

A discordant result is reported if:

- A slide is diagnosed as malaria negative by one reader and as malaria positive by a second reader or vice versa
- The species diagnosed by the two readers do not match,
- The number of species/slide differs between the two readers
- The parasite density of the lower reading is more than 10% lower than the higher reading. Example:

If: $(Reading_{High} – Reading_{Low}) / (Reading_{High} * 0.1) > 1$ => discordant result

If: $(Reading_{High} – Reading_{Low}) / (Reading_{High} * 0.1) \leq 1$ => congruent result

This formula only applies if the higher parasite count is ≥200 parasites / µL.

If both counts are <200 parasites / µL, this is considered a congruent result and does not require a further reading.

18.7 Informed consent and assent

Participant Information Sheet

Universitas Gadjah Mada   YPKMP   menzies school of health research

This is for you to keep

Lembaran ini untuk anda simpan

Protocol Title: A randomized controlled trial on malaria primaquine treatment in Timika, Indonesia (TRIPI)

Judul Protokol: Uji klinis Acak pengobatan malaria dengan primakuin di Timika, Indonesia (TRIPI)

Note: If you are a parent or guardian of a child below 18 years old, please read "you" as "your child".

Catatan: Jika anda orang tua atau wali anak usia dibawah 18 tahun, harap kata "anda" dibaca sebagai "anak anda"

You have been tested for malaria and the test shows that you have the disease. Malaria is a common infection in Papua-Indonesia. Young babies, as early as one day old as well as children and adults are affected. Those infected can suffer from a very sever course or in some cases may experience little to no discomfort at all.

In Timika, we have identified Dihydroartemisinin-Piperaquine (also known as the blue tablets) as a very good drug for malaria treatment in children and non-pregnant adults. The drug works very fast and has few known side effects.

People with tertian malaria, apart from the blue/green tablets also receive primaquine for 14 days to clear the remaining parasites in the liver. This is very important to prevent recurrent malaria.

We would like to improve malaria treatment and understand the best way to deliver treatment. We have good reason to believe that many people who suffer from malaria and are cured with the blue tablets also have tertian malaria. Soon after the first malaria episode these people will fall sick again with tertian malaria. We believe that this can be prevented if everybody receives primaquine. Primaquine needs to be given over the course of two weeks.

We would like to see whether this is best done in the presence of a medical person or can be done at home.

With this study we would like to address these two topics. The study result will inform the National Policy on how to improve the population health.

Hasil pemeriksaan darah anda menunjukkan anda terkena malaria. Malaria merupakan penyakit yang biasa terjadi di Papua-Indonesia. Risiko terkena malaria dimulai sejak lahir, juga pada usia anak-anak dan dewasa. Mereka yang terkena malaria dapat menjadi berat atau bahkan tanpa gejala sama sekali.

Di Timika, telah ditemukan obat anti malaria yang efektif: Dihydroartemisinin-piperaquine (juga dikenal sebagai tablet biru/hijau) yang merupakan obat yang baik untuk anak dan orang dewasa. Obat ini bekerja secara cepat dengan efek samping sedikit. Orang dengan malaria tertiana juga diberikan obat primakuin selama 14 hari untuk membersihkan sisa parasite di hati/liver. Ini sangat penting untuk mencegah kekambuhan.

Kami hendak menilai bagaimana agar pengobatan primakuin tersebut dapat berjalan dengan baik dan efektif. Hasil obsevasi kami menunjukkan bahwa orang yang terkena malaria dan sudah minum obat biru/hijau juga mengalami malaria tertiana. Segera setelah terkena malaria, dia akan kambuh dengan malaria tertiana. Kami percaya bahwa hal ini dapat dicegah dengan pemberian primakuin selama 14 hari untuk setiap jenis malaria. Kami ingin juga melihat apakah pemberian primakuin lebih baik diberikan dengan supervise oleh petugas kesehatan atau dapat dilakukan sendiri dirumah.

Dengan penelitian ini, kami ingin melihat kedua hal tersebut diatas. Hasil penelitian ini akan menjadi masukan bagi pengambil kebijakan dalam meningkatkan kesehatan masyarakat.

We offer you to participate in this study. If you participate you will be given the blue/green tablets for 3 days and primaquine for 14 days to clear up the remaining parasites in the liver. Primaquine cannot be given to people with low level of G6PD as it can destroy the red blood cells. We will therefore test your G6PD levels before proceeding. If you are female between the age of 14 and 49 we will also check if you are pregnant. This is a routine procedure and will be done in all women of the respective age, irrespective of marital status.
Kami menawarkan anda untuk ikut serta dalam penelitian ini. Jika anda ikut serta, kami akan memberikan tablet biru/hijau selama 3 hari dan primakuin selama 14 hari untuk

membersihkan sisa parasite dalam hati/liver. Primakuin tidak dapat diberikan pada orang yang kadar G6PD nya rendah, karena dapat menghancurkan sel darah merah. Oleh sebab itu kami akan melakukan pemeriksaan kadar G6PD sebelum memberikan primakuin. Jika anda wanita usia 14 dan 49 tahun kami juga akan melakukan pemeriksaan kehamilan. Prosedur ini rutin dilakukan pada wanita tanpa melihat status pernikahan.

If you agree to join the study, the following will happen:

1. We will perform a complete physical examination to ensure that you do not have severe malaria or suffer from other severe disease. If medical attention is required, we will refer you to clinic/hospital for further care.
2. We will collect blood from you. If you are above five years of age we will collect 5ml of blood from your arm, this is approximately the same as one teaspoon full. If you are below the age of six we will prick you in the finger or foot and collect approximately six drops of blood. Please note that blood collection is painful.
3. The blood will be checked for malaria, haemoglobin, drug concentration, G6PD level, and the level of immunity you have against malaria.
4. Your DNA (your inherited blueprint) contained in the blood you give will also be checked to see if you have any variations in the red blood molecules related to malaria such as lack of glucose-6-phosphate and other factors that may affect malaria treatment outcome.
5. The malaria parasite in your blood will also be examined.

Jika anda setuju untuk ikut serta, maka akan dilakukan hal-hal berikut:

1. *Kami akan melakukan pemeriksaan fisik lengkap untuk memastikan bahwa anda tidak mempunyai gejala malaria berat. Jika anda sakit berat, maka kami akan merujuk ke rumah sakit untuk penanganan selanjutnya.*
2. *Kami akan mengambil darah anda. Jika usia anda diatas 5 tahun, kami akan mengambil darah sebanyak 5 ml dari lengan anda yang kurang lebih sama dengan satu sendok makan. Jika anda berusia 5 tahun kebawah maka kami akan mengambil darah lewat jari sebanyak 6 tetes darah. Proses pengambilan ini akan menimbulkan rasa sakit dan akan hilang segera.*
3. *Darah anda akan dilakukan pemeriksaan malaria, Hb, kadar G6PD, kadar obat, dan kadar tingkat kekebalan terhadap malaria.*
4. *DNA yang terdapat pada darah anda akan memberikan informasi apakah terdapat variasi dari molekul sel darah merah terkait malaria misalnya jumlah G6PD yang rendah dan factor lain yang dapat mempengaruhi hasil pengobatan.*
5. *Parasit malaria didalam darah anda akan diperiksa juga.*

We will then give you treatment for your malaria illness, and see you each day for three days to make sure you feel better. By a lottery system we will then either give you a dose of more malaria treatment every day or you will receive the full treatment course and will be asked to take your daily course at home. You cannot chose how we will give you your treatment. We will ask you to come and see us again 2 weeks after you have started the primaquine course, 1 month later and then each month for a total of 6 months. We will give you a call to see how you are doing three, four and five months later and will ask you to come and see us again six months after you have been treated. Whenever you believe you have a fever we also ask you to come back. Whenever you come and see us we will collect six drops of blood from you and do a physical exam.

Kami juga akan memberikan pengobatan untuk malaria dan akan bertemu dengan anda selama 3 hari berturut-turut untuk memastikan anda merasakan perbaikan. Kami akan mengundi apakah anda mendapatkan pemberian primakuin dan diminum di rumah atau dalam pengawasan petugas kesehatan. Anda tidak dapat memilih kelompok pengobatan tersebut. Kami akan meminta untk datang kembali 2 minggu setelah pemberian primakuin dimulai, 1 bulan kemudian dan selanjutnya setiap bulan sampai total 6 bulan. Kami akan menelpon untuk menanyakan keadaan anda 3,4 dan 5 bulan setelah pengobatan dan meminta anda datang pada bulan ke 6 setelah terapi. Setiap saat anda merasa panas, anda dapat memberitahu kami. Pada setiap kunjungan tersebut, kami akan mengambil 6 tetes darah dari jari/tumit dan melakukan pemeriksaan fisik.

Benefits of these Studies: Being part of this study will ensure that if you get malaria this will be diagnosed quickly and you are given a good treatment against malaria. You will not be charged any money for your malaria treatment.

If you do not wish to participate in these studies it will NOT affect your right to receive standard health care administered at this clinic.

Keuntungan dari Penelitian ini: *Dengan berpartisipasi pada penelitian ini maka anda akan menerima penanganan malaria yang baik. Anda tidak perlu membayar biaya apapun selama penelitian berlangsung..*

Jika anda tidak ingin berpartisipasi dalam penelitian ini, anda akan tetap mendapatkan penanganan sesuai standard pelayanan.

You can withdraw your consent from the study and still receive the treatment as you would normally.

If you have any questions about this study, you may contact Dr Rini on 0811491699 or the study doctors in this clinic.

In case of an emergency you should return to this clinic or if it is after hours present yourself to the RSUD Kabupaten Mimika or RS Mitra Masyarakat Emergency Room and inform the doctor that you have been a participant in this study.

If you have a complaint about the study then these should be addressed to Dr. Rini at RSUD Kabupaten MImika

If you wish to know what this study showed: - you can write to Dr. Rini at RSUD Kabupaten Mimika. There will be no name or other patient identification in any study report which will be published later on.

Anda dapat mengundurkan diri dari penelitian setiap saat dan masih akan tetap menerima perawatan standard.

Jika anda mempunyai pertanyaan, *dapat menghubungi Dr. Rini di 0811491699 atau dokter penelitian di lapangan.*

Pada keadaan darurat, *anda harus segera membawa bayi anda ke Unit Gawat Darurat di RSUD Kabupaten Mimika atau RS Mitra Masayarakat dan memebritahu dokter yang bertugas bahwa bayi anda ikut dalam penelitian.*

Jika ada keluhan *mengenai penelitian dapat menguhubungi Dr. Rini di RSUD Kabupaten Mimika*

Jika anda ingin mengetahui hasil penelitian: *anda dapat menulis ke Dr. Rini di RSUD Kabupaten Mimika. Kami tidak akan menyebut nama pasien pada setiap hasil penelitian yang akan di publikasi kemudian.*

CONSENT FORM

Consent Form for the trial

A randomized controlled trial on malaria primaquine treatment in Timika, Indonesia (TRIPI)

Uji klinis Acak pengobatan malaria dengan primakuin di Timika, Indonesia (TRIPI)

<u>This form means I can say “No”</u>
Form ini berarti saya dapat mengatakan “Tidak”

I have read and understood the information sheets attached, and have been given the opportunity to discuss the study and ask questions.

I agree that my samples can be used in the research outlined on the information sheet.

I understand that I do not have to participate in this study.

If I do not wish participate in the study I will still receive the standard health care administered at this clinic.

Saya telah membaca dan memahami informasi pada lembaran terlampir dan telah diberikan kesempatan untuk mendiskusikan dan menanyakan.

Saya setuju bahwa sampel saya dapat digunakan untuk penelitian seperti yang tercantum dalam lembar informasi.

Saya memahami bahwa saya tidak harus berpartisipasi pada penelitian ini.

Jika saya tidak berpartisipasi pada penelitian inisayaa masih akan menerima pelayanan kesehatan standard.

I …………………………………………………… agree to be enrolled in the above named study. As a participant in the study I agree to have regular blood samples collected. I understand that my stored samples will not be used for any other purpose outside of this study.

I am aware that I can withdraw my consent at any time of my own choosing.

Saya (orang tua/pengasuh dari)…………………… setuju untuk ikut serta dalam penelitian tersebut diatas. Sebagai perserta penelitian saya setuju untuk diambil darah secara teratur selama follow up. Saya memahami bahwa sampel saya tidak akan digunakan untuk kepentingan lain.

Saya memahami bahwa saya dapat mengundurkan diri dari penelitian setiap saat.

Signature : ……………………………………….

Date : ……………………………………….

Witness Name : ……………………………………….

Signature : …………………………………………..

Tanda tangan (orang tua) **:** *………………………………………….*

Tanggal **:** *………………………………………….*

Saksi **:** *………………………………………….*

Tanda tangan **:** *………………………………………….*

ASSENT FORM

Assent Form for the trial:

A randomized controlled trial on malaria primaquine treatment in Timika, Indonesia (TRIPI)

Uji klinis Acak pengobatan malaria dengan primakuin di Timika, Indonesia (TRIPI)

This form means I can say "No"

Form ini berarti saya dapat mengatakan "Tidak"

Hello,

You have been diagnosed with malaria. Malaria is a very serious disease, but you will receive good treatment in a second. We are trying to improve the treatment for malaria by understanding whether it is better to see a doctor for treatment daily or not and if treatment outcome acan be improved by giving everybody the same treatment.

We therefore want to invite you to take part in this study. If you agree we will collect some blood from your arm and do a physical exam. We will then either ask you to come and see us for daily treatment the next 2.5 weeks or ask you to come back in two weeks time only. This depends on a selection system similar to a lottery. We will also ask you to come back monthly one, and two months after you have been treated for malaria and half a year later. We will call you three, four and ficve months after you have received malaria treatment to see how you are doing. Whenevr you feel like you are having a fever we also ask you to come back, so we can make sure you don't have malaria. Every time you come and see us we will collect up to six drops of blood from you.

You don't have to participate. You will be treated for malaria just as well ad nobody will be angry if youdon't want to participate. You can also later always say you don't want to participate and this will not have any disadvantage for you.

Do you have any questions?

If you want to participate please sign below.

Anda telah didiagnosis sebagai malaria. Malaria adalah penyakit yang serius, namun anda akan segera mendapatkan obat yang bagus. Kami sedang berupaya untuk meningkatkan kualitas pengobatan malaria dengan cara melihat apakah pengobatan akan lebih baik jika diawasi oleh tenaga kesehatan.

Kami mengundang anda untuk ikut serta dalam penelitian ini. Jika anda setuju, kami akan mengambil darah dari lengan anda dan melakukan pemeriksaan fisik. Kami kemudian akan meminta anda untuk datang setiap hari selama 14 hari atau meminta anda datang pada hari ke 14 saja. Pembagian kelompok ini akan diundi.

Kami akan meminta untk datang kembali 1 bulan kemudian dan selanjutnya setiap bulan sampai total 6 bulan. Kami akan menelpon untuk menanyakan keadaan anda 3,4 dan 5 bulan setelah pengobatan dan meminta anda datang pada bulan ke 6 setelah terapi. Setiap saat anda merasa pansa, anda dapat memberitahu kami. Pada setiap kunjungan tersebut, kami akan mengambil 6 tetes darah dari jari/tumit dan melakukan pemeriksaan fisik.

Anda tidak harus untuk ikut serta. Anda akan tetap diberikan pengobatan malaria sesuai protocol. Jika anda setuju ikut serta, anda dapat sewaktu-waktu mengundurkan diri dari penelitian dan tidak akan mempengaruhi pengobatan yang diberikan.
Apakah ada pertanyaan?

Jika anda ingin ikut serta, silahkan untuk menandatangani dibawah ini:

Signature : …………………………………………
Date : …………………………………………
Witness Name : …………………………………………
Signature : …………………………………………

Tanda tangan : ……………………………………………
Tanggal : ……………………………………………
Saksi : ……………………………………………
Tanda tangan : ……………………………………………

Participant information / verbal consent text: sheet patients and community members

This is for you to keep

***Protocol Title:** A randomized controlled trial on malaria primaquine treatment in Timika, Indonesia (TRIPI)*

Note: If you are a parent or guardian of a child below 18 years old, please read "you" as "your child".

We are currently conducting a study on malaria treatment (the TRIPI study) conducted by the University of Gadjah Mada, Indonesia, YPKMP, Indonesia and the Menzies School of Health Research, Australia. As part of this study we would like to understand your views on malaria, where you would seek treatment, what treatment you would take and how a malaria infection affects your life.

We would like to discuss this with you in an interview. The interview will take approximately one hour and will be recorded.

We will want to publish an article on your and other opinions on malaria and malaria treatment. We will do so in a way that nobody will know that you participated. We will also not tell anybody that you participated or what you said.

You can refuse to give us an interview. You can do so now or at any stage of the interview. If you change your mind during the interview just let us know, we will delete the entire interview and we will not contact you again. We do not ask you to sign anything, however ask you to agree in words to the interview once the tape recorder is switched on.

Should you have any questions or complaints about the interview now or later, please feel free to ask the interviewer or contact:

Dr Rini on 0811491699 at RSUD Kabupaten Mimika

If you agree to participate, please say “I agree” once I have switched on the tape recorded.

Participant information / verbal consent text : health care providers

This is for you to keep

Protocol Title: ***A randomized controlled trial on malaria primaquine treatment in Timika, Indonesia (TRIPI)***

Note: If you are a parent or guardian of a child below 18 years old, please read "you" as "your child".

We are currently conducting a study on malaria treatment (the TRIPI study) conducted by the University of Gadjah Mada, Indonesia, YPKMP, Indonesia and the Menzies School of Health Research, Australia. As part of this study we would like to understand your views on malaria, different malaria treatment options and pre-treatment procedures you deem necessary to deliver safe malaria treatment. We would like to discuss this with you in an interview. The interview will take approximately one hour and will be recorded.

We will want to publish an article on your and other opinions on the above topics. We will do so in a way that nobody will know that you participated. Your participation is confidential. You can refuse to give us an interview. You can do so now or at any stage of the interview. If you change your mind during the interview, just let us know, we will delete the entire interview and we will not contact you again. We do not ask you to sign anything, however ask you to agree in words to the interview once the tape recorder is switched on.

Should you have any questions about the interview now or later, please feel free to ask the interviewer or contact:

Dr Rini on 0811491699 at RSUD Kabupaten Mimika

If you agree to participate, please say “I agree” once I have switched on the tape recorded.
